# The Nuclear Localization of ACLY Guards Early Embryo Development Through Recruiting P300 and HAT1 to Promote Histone Acetylation and Transcription

**DOI:** 10.1002/advs.202414367

**Published:** 2025-06-05

**Authors:** Yerong Ma, Yingyi Zhang, Weijie Yang, Xiaomei Tong, Siya Liu, Yan Zhou, Mengjia Qiu, Huifang Jiang, Zhanhong Hu, Peipei Ren, Yan Rong, Mengru Lai, Jiamin Jin, Fei Huang, Liujian Ouyang, Feng Zhou, Heng‐Yu Fan, Yin‐Li Zhang, Songying Zhang

**Affiliations:** ^1^ Assisted Reproduction Unit, Department of Obstetrics and Gynecology, Sir Run Run Shaw Hospital, School of Medicine, Zhejiang University, Zhejiang Key Laboratory of Precise Protection and Promotion of Fertility Zhejiang Provincial Clinical Research Center for Reproductive Health and Disease Hangzhou 310016 P. R. China; ^2^ MOE Key Laboratory of Biosystems Homeostasis and Protection and Zhejiang Provincial Key Laboratory for Cancer Molecular Cell Biology Life Sciences Institute Zhejiang University Hangzhou Zhejiang 310058 P. R. China; ^3^ Department of Endocrinology, Children's Hospital of Zhejiang University School of Medicine National Clinical Research Center for Child Health Hangzhou 310051 P. R. China

**Keywords:** ACLY, P300, HAT1, early embryo development, histone acetylation

## Abstract

Metabolic processes and epigenetic reprogramming are intricately interconnected; however, their mechanistic interplay remains unclear. This study elucidates the role of ATP‐citrate lyase (ACLY), an essential enzyme in acetyl‐CoA production that uniquely localizes to the nucleus in oocytes and early embryos. Maternal *Acly* deletion in oocytes preserves fertility due to the compensatory upregulation of Acetyl‐CoA Synthetase 2 (ACSS2), whereas zygotic *Acly* knockout causes developmental arrest at the pre‐blastocyst stage without ACSS2 induction. Mechanistically, nuclear ACLY recruits and interacts with histone acetyltransferases, specifically E1A binding protein p300 (P300) and histone acetyltransferase 1 (HAT1), supplying acetyl‐CoA for histone acetylation to activate transcription, which is essential for embryogenesis. Clinically, enhanced ACLY nuclear localization correlates with superior quality of human embryos. Functionally, AKT‐mediated phosphorylation (Thr447/Ser451/Ser455) drives the nuclear translocation of ACLY and facilitates its interaction with HAT1 and P300. Inhibition of ACLY or its phosphorylation disrupts the promoting effects of AKT activators, such as insulin‐like growth factor‐1 (IGF‐1), on blastocyst formation. These findings suggest that ACLY is a metabolic hub that bridges signaling and epigenetic remodeling, ensuring acetyl‐CoA availability for chromatin modifications, and offering insights into the metabolic determinants of embryo viability and potential therapeutic targets for infertility.

## Graphical abstract

ACLY is vital for early embryo development. IGF‐1 activates AKT to phosphorylate ACLY, driving its nuclear localization and recruitment of HATs (P300/HAT1), boosting acetyl‐CoA production and histone acetylation for transcriptional activation. Conversely, ACLY deficiency (via knockdown, knockout, or AKT inhibition) reduces nuclear acetyl‐CoA, disrupts HAT activity, and triggers embryonic arrest through transcriptional silencing.

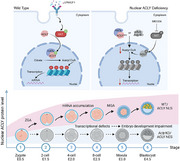

## 1 Introduction

After fertilization, mammalian preimplantation embryo development involves several cleavage divisions and the first cell fate determination, leading to the formation of a blastocyst comprising the inner cell mass (ICM) and trophectoderm (TE).^[^ ^1^, ^2^, ^3^ ^]^ During this period, a critical process termed maternal‐to‐zygotic transition (MZT) orchestrates embryo development, involving the clearance of maternal RNA and proteins and zygotic genome activation (ZGA).^[^ ^4^, ^5^, ^6^ ^]^ After ZGA, transcripts activated at 8‐cell (also called mid‐preimplantation gene activation, MGA) contribute to cell lineage specification in mice and humans.^[^ ^7^, ^8^ ^]^ Several studies have demonstrated that ZGA activation and epigenetic remodeling are driven by dynamic metabolic changes.^[^ ^9^, ^10^, ^11^, ^12^ ^]^ However, whether metabolism controls cell lineage specification is inadequately understood, and the specific metabolic enzymes responsible for controlling epigenetic modifications and gene expression remain unclear.

Early mammalian embryogenesis is dynamically regulated through metabolic‐epigenetic crosstalk, in which nutritional support from the intrinsic and extrinsic microenvironments coordinates developmental progression. Initially dependent on pyruvate/lactate metabolism, embryos undergo a glucose‐dependent metabolic shift postmorula, a transition that is conserved across humans and mice.^[^ ^13^, ^14^, ^15^, ^16^ ^]^ These metabolic fluxes are mechanistically coupled to epigenetic remodeling, with key intermediates (α‐KG, S‐adenosylmethionine, L‐2‐HG) modulating histone methylation (H3K27me3, H3K4me3) and DNA methylation to regulate pluripotency and blastulation.^[^ ^16^, ^17^, ^18^, ^19^, ^20^, ^21^, ^22^ ^]^ Pyruvate‐derived mitochondrial acetyl‐CoA synthesis, enhanced by nuclear translocation of TCA cycle enzymes, not only fuels ZGA but also sustains pluripotency through localized energy provision.^[^ ^9^, ^23^ ^]^ Central to this regulatory axis, histone acetyltransferases (HATs) such as E1A binding protein p300 (P300) serve as metabolic‐epigenetic integrators: P300 orchestrates enhancer activation via H3K27ac deposition, and non‐catalytically primes transcriptional competence.^[^ ^24^ ^]^ Crucially, nuclear acetyl‐CoA pools, modulated by the glucose/glutamine/acetate flux, act as metabolic rheostats governing H3K27ac dynamics to balance lineage bifurcation.^[^ ^25^, ^26^, ^27^, ^28^ ^]^ Advanced multiomics approaches have delineated metabolic control over histone acetylation. Key questions persist regarding the spatiotemporal regulation of HATs (e.g., P300 nuclear dynamics during lineage commitment) and the identity of post‐ZGA enzymes that bridge metabolites to transcriptional outputs.

Central to this axis is acetyl‐CoA, which links nutrient availability to protein acetylation, and provides acetyl groups for HATs, thereby influencing histone acetylation and gene transcription.^[^ ^29^, ^30^, ^31^, ^32^, ^33^, ^34^ ^]^ The synthesis of acetyl‐CoA depends on two key enzymes, ATP‐citrate lyase (ACLY) and acyl‐CoA synthetase short‐chain family member 2 (ACSS2).^[^ ^35^, ^36^, ^37^ ^]^ In somatic cells, ACLY and ACSS2 exhibit functional redundancy and context‐dependent dominance.^[^ ^38^, ^39^ ^]^ ACLY is highly expressed in several tumor cells and converts mitochondrially derived citrate into acetyl‐CoA,^[^ ^35^, ^40^, ^41^ ^]^ whereas ACSS2 directly activates acetate as an alternative acetyl‐CoA source under nutrient stress.^[^ ^42^, ^43^, ^44^ ^]^ Both enzymes are regulated by post‐translational modifications; ACLY undergoes AKT‐mediated phosphorylation to enhance nuclear retention,^[^ ^45^, ^46^ ^]^ whereas ACSS2 is transcriptionally induced during hypoxia or low‐glucose conditions.^[^ ^42^, ^47^ ^]^ Despite their established roles in cancer and somatic stem cells, their functions during the MZT remain unknown. Strikingly, global *Acly* knockout causes embryonic lethality at E8.5,^[^ ^48^ ^]^ whereas *Acss2* deficiency is tolerated in early embryos.^[^ ^44^ ^]^ This reflects ACLY's unique role of ACLY during early embryogenesis. Moreover, the functions of maternal and zygotic ACLY and ACSS2 remain unclear.

Here, we use oocyte‐specific (*Acly* ^flox/flox^;*Gdf9*‐Cre) and global (*Acly* ^−/−^) knockout models to elucidate the mechanism of ACLY in coordination of metabolism, histone acetylation, and transcription during MZT, and to examine the collaborative roles of ACLY and ACSS2. We demonstrated that nuclear ACLY catalyzes acetyl‐CoA production and recruits P300 and histone acetyltransferase 1 (HAT1) to the nucleus to promote histone acetylation at pluripotency loci in an AKT phosphorylation‐dependent manner. We also found partially redundant roles of ACLY and ACSS2 during oogenesis and early embryonic development. This study established ACLY as a critical metabolic‐epigenetic integrator that translates microenvironmental cues into cell fate decisions during early embryonic development.

## 2 Results

### 2.1 Increases in Histone H3 Acetylation Are Concomitant with Enhanced Citrate Metabolism with Embryo Development From 8‐Cell to Blastocyst

**Figure 1:**
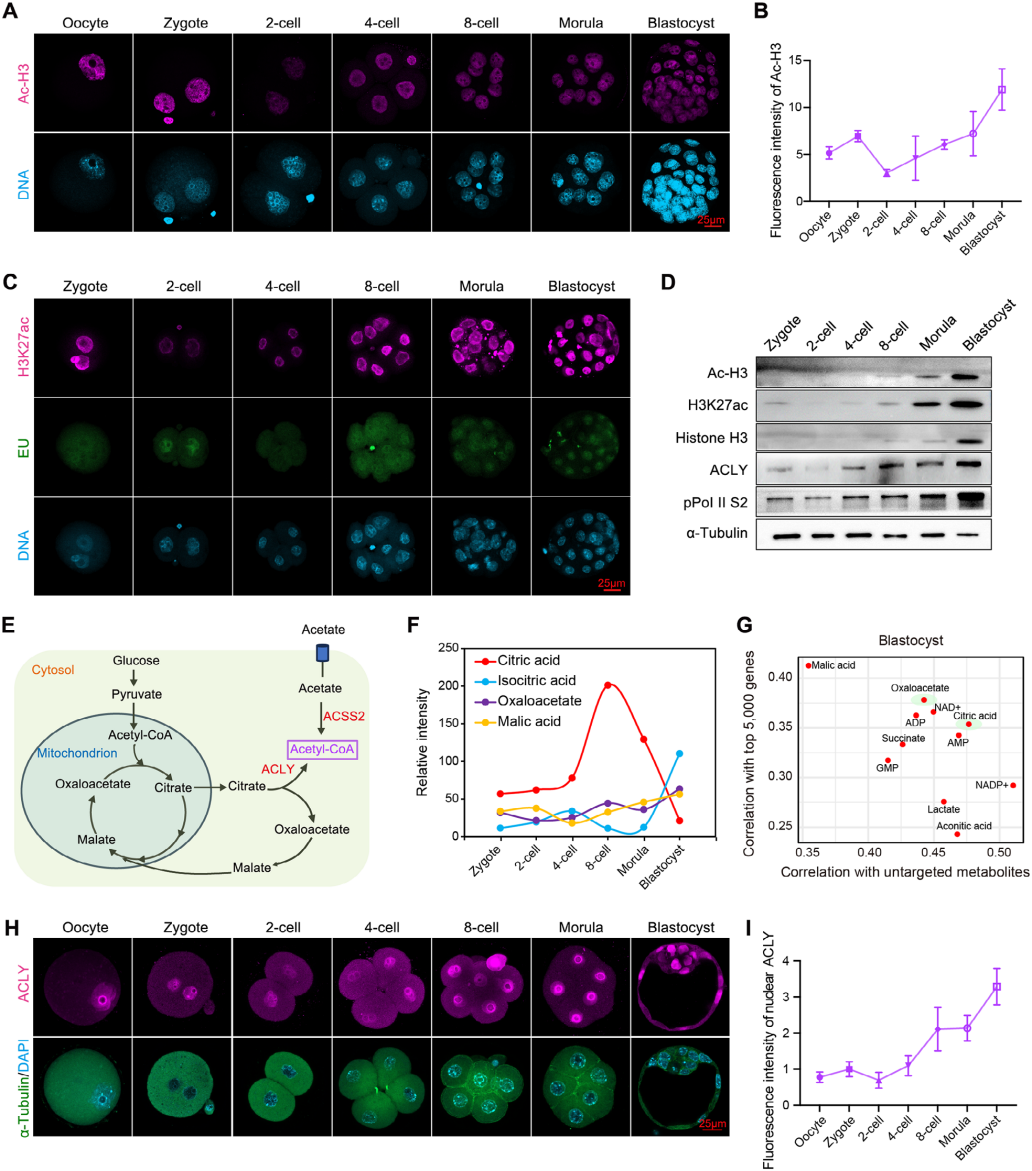
Stage‐specific histone acetylation increase correlates with citrate metabolism during embryogenesis. (A,B) Representative images (A) and quantitative analysis (B) of mouse oocytes and embryos at different stages stained with anti‐Ac‐H3 (violet) antibody (n = 16 oocytes/embryos per group). Nuclei were stained with DAPI (light blue). Scale bar = 25 µm. (C) Representative images of mouse embryos at different stages stained with anti‐ac‐H3K27ac (violet) antibody and EU staining (green) with nuclei stained with DAPI (light blue). Scale bar = 25 µm. (D) Western blot results of 200 mouse embryos at different stages (n = 2). α‐Tubulin served as a loading control. (E) Schematic diagram of ACLY and ACSS2 involvement in the physiological process of acetyl‐CoA production. (F) Changes in the content of several metabolites in early embryos at different stages. (G) The correlation analysis of top 5000 transcription active genes with untargeted metabolites at blastocyst stage in mouse. (H,I) Representative images (H) and quantitative analysis (I) of mouse oocytes and embryos at different stages stained with anti‐ACLY (violet) and anti‐α‐Tubulin (green) antibodies (n = 30). The fluorescence intensity of ACLY reflects the intensity in the embryo nuclei. Nuclei were stained with DAPI (light blue). Scale bar = 25 µm. Data are presented as mean ± S.D.

The dynamics of histone acetylation during early embryonic development have been extensively investigated^[^ ^49^ ^]^; however, the metabolic mechanism underlying histone acetylation during this period has not been extensively studied. Using immunofluorescence (IF) and western blot (WB) analyses, we found that the levels of histone H3 pan‐acetylation (pan Ac‐H3) and H3K27 acetylation (H3K27ac) were low in zygote, 2‐cell, and 4‐cell embryos and dramatically increased from the 8‐cell stage to the blastocyst stage (**Figure** **1**A–D, Figure S1A), Supporting Information. This pattern correlated with escalating transcriptional activity, as evidenced by the incorporation of EU‐labeled nascent RNA (Figure 1C) and the phosphorylation of the RNA polymerase II C‐terminal domain at serine 2 (pPol II S2, Figure 1D).

Given that cytosolic acetyl‐CoA can be generated from endogenous citrate or exogenous acetate (Figure 1E), we focused on identifying potential metabolites contributing to the heightened demand for acetyl‐CoA in 8‐cell to blastocyst embryos. Using reported metabolic data,^[^ ^50^ ^]^ we noticed that the citrate cycle was highly enriched after ZGA treatment. Notably, citrate levels peaked at the 8‐cell stage and were gradually metabolized, and contrasting trends of isocitrate were observed (Figure 1F). Concurrently, the levels of other citrate cycle products such as oxaloacetate and malate gradually increased from the 8‐cell to the blastocyst stage (Figure 1F), aligning with the heightened metabolism of the citrate cycle and its correlation with histone acetylation during early embryo development. We conducted correlation analyses of each metabolite with other metabolites and the top 5000 genes of 8‐cell embryos or blastocysts by integrating metabolic and RNA‐seq data, as previously reported.^[^ ^50^, ^51^ ^]^ Furthermore, the results revealed stage‐specific enrichment of citrate cycle metabolites (e.g., citrate and oxaloacetate) from the 8‐cell to blastocyst stages (Figure 1G and Figure S1B). The results demonstrate a close association between citrate cycle metabolites and gene expression from 8‐cell to blastocyst development and indicate that enhanced citrate cycle flux co‐occurs with histone acetylation during early embryo development.

### 2.2 ACLY Is Highly Localized in the Nuclei of Mouse Oocytes and Early Embryos

We then investigated the roles of ACLY and ACSS2, the two key enzymes responsible for acetyl‐CoA production during early embryonic development. Analysis of published RNA‐seq data (E‐MTAB‐2950) from mouse oocytes and embryos^[^ ^52^ ^]^ revealed markedly higher *Acly* mRNA levels than *Acss2* across all early embryonic stages (Figure S1C, Supporting Information). RT‐qPCR further confirmed the stage‐dependent upregulation of *Acly* expression from the 8‐cell to morula stage, in sharp contrast to the consistently low *Acss2* expression (Figure S1D, Supporting Information).

Consistent with a previous study,^[^ ^44^ ^]^ we also found that ACSS2 depletion did not affect early embryo development because si*Acss2*‐treated zygotes and ACSS2‐IN2 inhibitor‐exposed embryos showed normal blastocyst formation potential (Figure S1E‐F). Importantly, zygotic *Acss2* knockdown did not induce the compensatory upregulation of ACLY, whereas global H3K27ac levels remained stable (Figure S1G–I, Supporting Information).

We examined the expression and role of ACLY in early embryogenesis. Consistent with *Acly* mRNA profiles, WB and IF analyses demonstrated progressive accumulation of the ACLY protein from the zygote to the blastocyst stage (Figure 1D,H‐I). Intriguingly, ACLY exhibited exclusive nuclear localization in oocytes and early embryos (Figure 1H), diametrically opposed to its cytoplasmic presence in somatic cells, such as HeLa cells, granulosa cells, and endometrial glandular epithelium (Figure S1J, Supporting Information). Temporally, ACLY upregulation from the 8‐cell stage onward strongly correlated with the acquisition of histone H3 acetylation patterns (Figure 1A–D), suggesting that ACLY is a potential regulator of histone acetylation during early embryo development.

### 2.3 *Acly*‐Null Embryos Are Mostly Lethal Before the Blastocyst Stage

**Figure 2:**
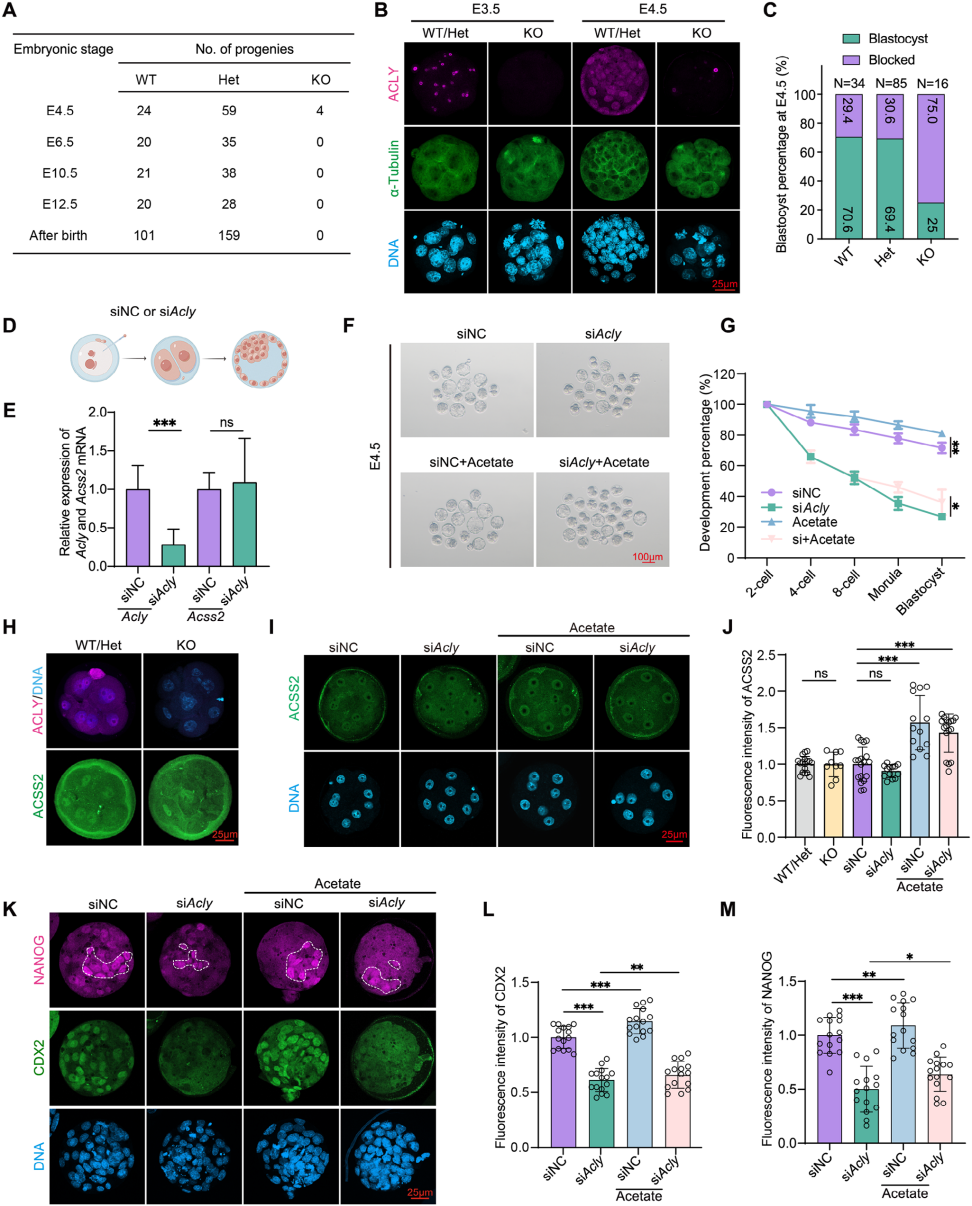
Zygotic *Acly* is required for embryogenesis, and ACLY exhibits partial functional redundancy with ACSS2 in the presence of acetate in vitro. (A) Genotypes of embryos collected at different time points after mating of heterozygous (*Acly* ^+/−^) mice. (B) Representative images of E3.5 and E4.5 embryos obtained from mating heterozygous (*Acly* ^+/−^) mice with immunofluorescence staining for ACLY (violet) and α‐Tubulin (green) (n = 50, WT/Het; n = 10, KO). Nuclei were stained with DAPI (light blue). Scale bar = 25 µm. (C) The proportion of blocked embryos and blastocysts at E4.5 for different genotypes (n = 3). (D) Diagram of the zygote microinjection process. (E) RT‒qPCR results of *Acly* and *Acss2* expression in 30 morulae developed from zygotes after injection with siNC or si*Acly*, normalized to *Actin* and *Gapdh* (n = 6). (F,G) The development process (F) and development percentage (G) at each stage for the embryos that developed from zygotes injected with 20 µM siNC or si*Acly*, with or without 2 µM acetate (n = 56, siNC; n = 60, si*Acly*; n = 60, Acetate; n = 58, si*Acly+*Acetate). Scale bar = 100 µm. (H) Representative images of 8‐cell stage embryos obtained from mating heterozygous (*Acly* ^+/−^) mice with immunofluorescence staining for ACLY (violet) and ACSS2 (green) (n = 17, WT/Het; n = 9, KO). Nuclei were stained with DAPI (light blue). Scale bar = 25 µm. (I) Representative images of 8‐cell embryos stained with anti‐ACSS2 (green) after treatment (n = 18). Nuclei were stained with DAPI (light blue). Scale bars = 25 µm. (J) Statistical analysis of the fluorescence intensity of ACSS2 in *Acly*‐KO or knocking down embryos cultured with or without 2 µM acetate (n = 17, WT/Het; n = 9, KO; n = 18, siNC; n = 18, si*Acly*; n = 18, acetate; n = 18, si*Acly*+acetate). Data are presented as mean ± S.D. (K‐M) Representative images (K) and qualitative analysis (L‐M) of blastocysts stained with anti‐NANOG (violet) antibody and anti‐CDX2 (green) antibody after treatment (n = 15). The highlighted areas were inner cell mass. Nuclei were stained with DAPI (light blue). Scale bar = 25 µm. Data are presented as mean ± S.D. *NS p* > 0.05; * *p* < 0.05; ** *p* < 0.01, *** *p* < 0.001. Student's t test (E, G, and J). One‐way ANOVA (J, L, and M).

To elucidate the effects of zygotic *Acly* deletion on embryo development, we generated *Acly* oocyte‐specific knockout mice (*Acly^flox/flox^; Gdf9‐Cre*, *Acly* cKO) by crossing *Acly^flox/flox^* mice with *Gdf9‐Cre* mice (Figure S1K,L, Supporting Information) and further generated *Acly* knockout (KO) mice, wherein the *Acly* KO allele lacked exons 3–6 (Figure S1K,M, Supporting Information) and was verified by genotyping (Figure S1N, Supporting Information). Heterozygous (*Acly* ^+/–^) male and female mice were crossed, but no homozygous (*Acly* ^–/–^) *Acly* KO mice were obtained after 6 months (**Figure** **2**A). Subsequently, we conducted single‐embryo genotyping and IF across different developmental stages to ascertain the timing of embryonic lethality. Among the 87 blastocysts genotyped from six litters of *Acly* heterozygous KO mice at embryonic day 4.5 (E4.5), we identified 24 wild‐type (*Acly* ^+/+^), 59 heterozygous KO (*Acly* ^+/–^), and 4 homozygous KO (*Acly* ^–/–^) embryos, with the latter significantly deviating from the expected 1:2:1 Mendelian ratio (Figure 2A). Notably, only 4.6% (4 in 87 embryos) of E4.5 blastocysts were *Acly* ^–/–^ embryos (Figure 2A). IF analysis revealed that numerous *Acly* ^–/–^ embryos were developmentally arrested at earlier stages (Figure 2B). An analysis of embryos collected at E2, E2.5, and E4 revealed that the formation of 2‐cell and 4‐cell stage embryos was slightly affected, whereas the formation of 8‐cell stage and blastocyst embryos was significantly affected (Figure S1O). Further analysis at E4.5 showed that 70.6% of *Acly* ^+/+^ and 69.4% of *Acly* ^+/–^ embryos developed into blastocysts, in stark contrast to only 25% of *Acly* ^–/–^ embryos achieving this stage (Figure 2C). Genotyping of post‐implantation embryos revealed the absence of *Acly* ^–/–^ embryos at E6.5, E8.5, and E12.5, indicating that *Acly*‐null embryos predominantly failed to progress beyond the blastocyst stage and terminated development prior to E6.5 (Figure 2A).

### 2.4 Zygotic *Acly* Deletion Inhibits Cell Proliferation and Blastocyst Formation, but Hardly Induces DNA Damage or Apoptosis

To mechanistically dissect ACLY's role in embryogenesis, we employed two approaches: siRNA‐mediated knockdown and pharmacological inhibition using SB204990, a selective ACLY inhibitor. Zygotes were microinjected with either a negative control siRNA (siNC) or *Acly*‐targeting siRNA (si*Acly*) and cultured to the blastocyst stage (Figure 2D). The efficiency of *Acly* knockdown at mRNAlevels was verified by RT‒ RT‐qPCR. We observed a reduction in *Acly* expression relative to that in the siNC group (Figure 2E), whereas the mRNA and protein levels of ACSS2 remained comparable in both *Acly*‐KD and ‐KO embryos (Figure 2E, Figure 2H–J). *Acly* knockdown at the zygotic stage significantly delayed embryonic division, yielding a 2/3 reduction in the blastocyst formation rate compared with that in controls (Figure 2F,G). Moreover, upon microinjecting siRNAs (siNC or si*Acly*) in conjunction with *mCherry* mRNAs into a single blastomere at the 2‐cell stage (Figure S2A, Supporting Information), we observed compromised blastomere proliferation and significantly reduced blastocyst formation in the si*Acly* group (Figure S2B–D, Supporting Information). Similarly, treatment with SB204990 adversely affected early embryonic development, including blastocyst formation and blastomere number per blastocyst (Figure S2E–G, Supporting Information). Assessment of γ‐H2AX and TUNEL signals in control and si*Acly* embryos revealed that *Acly* depletion did not induce DNA damage and apoptosis (Figure S2H–K, Supporting Information).

During the 8‐cell to morula transition, embryonic blastomeres undergo lineage specification into two distinct populations: inner cell mass (ICM) and trophectoderm (TE) lineages. CDX2 is exclusively expressed in blastomeres committing to the TE lineage, while NANOG marks the ICM.^[^ ^1^, ^53^ ^]^ Single‐embryo RT‐qPCR revealed a pronounced decrease in both *Cdx2* and *Nanog* mRNA in *Acly* KO embryos compared to WT 8‐cell stage embryos (Figure S2L,M, Supporting Information). A concomitant reduction in CDX2 and NANOG levels was evident in morula embryos of the *Acly* KO group (Figure S2O–Q, Supporting Information) and in blastocysts from the si*Acly* group (Figure 2K–M). Notably, NANOG fluorescence intensity was diminished, with fewer NANOG‐positive blastomeres than those in the control group (Figure 2K–M and Figure S2O–Q, Supporting Information). These results indicated that zygotic ACLY deletion impaired the expression of cell lineage genes. Intriguingly, supplementing KSOM with 2 mM acetate partially rescued the developmental defects in *Acly*‐KD early embryos, including blastocyst formation and cell lineage differentiation (Figure 2F,G, K–M), with the upregulation of ACSS2 (Figure 2I,J). Given ACSS2's dual capacity to synthesize both acetyl‐CoA and crotonyl‐CoA, which serve as catalytic substrates for HATs, such as P300, to mediate both protein acetylation and crotonylation is shown in ref. ^[^ ^54^ ^]^. This bifunctional metabolic node critically regulates endoderm differentiation through coordinated epigenetic reprogramming [55]; therefore, we assessed global crotonylation (pan‐Cr) levels, which also enhanced pan‐Cr levels in both control and *Acly*‐KD embryos (Figure S3A,B, Supporting Information). These results indicate the indispensable role of ACLY in early embryonic development and partial functional redundancy between ACLY and ACSS2.

### 2.5 Zygotic ACLY Loss Leads to a decrease in Acetyl‐CoA, Histone Acetylation, and Transcriptional Activity

**Figure 3:**
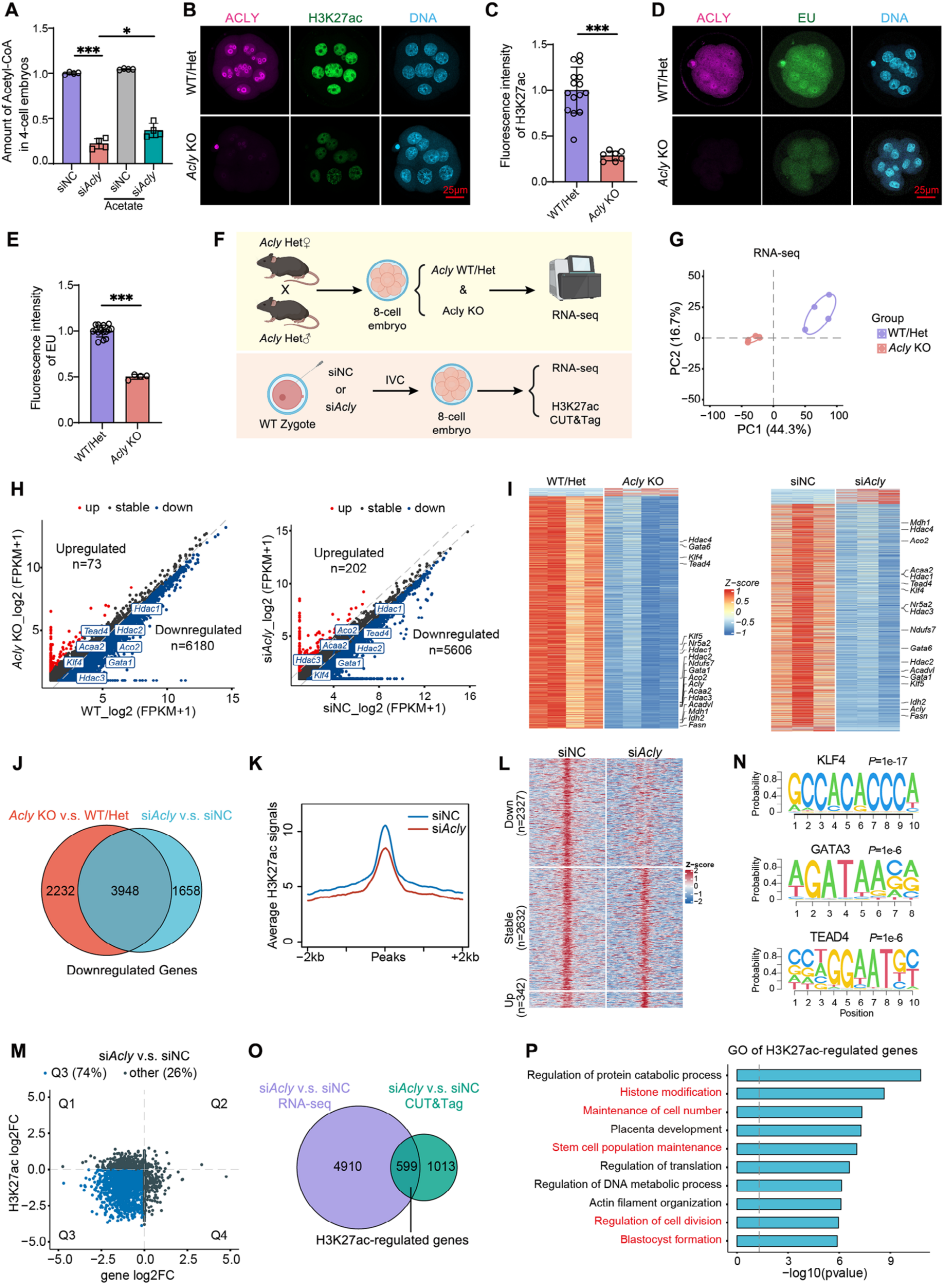
Zygotic ACLY loss leads to a decrease in acetyl‐CoA, histone acetylation, and transcriptional activity. (A) Statistical analyses of acetyl‐CoA content in all 500 4‐cell embryos after injecting siNC or si*Acly* at the zygote stage and cultured in KSOM with or without 2 mM acetate (n = 4). (B) Representative images of 8‐cell embryos obtained from mating heterozygous (*Acly* ^+/−^) mice, stained with anti‐ACLY (violet) antibody and anti‐H3K27ac (green) antibody. Nuclei were co‐stained with DAPI (light blue). Scale bar = 25 µm. (C) Quantitative analysis of the fluorescence intensity of H3K27ac using ImageJ (n = 25, WT/Het; n = 6, KO). (D‐E) Representative images (D) and quantitative analysis (E) of 8‐cell stage embryos obtained from mating heterozygous (*Acly* ^+/−^) mice, stained with anti‐ACLY (violet) antibody and EU (green) to evaluate the level of transcription (n = 15, WT/Het; n = 4, KO). Nuclei were stained with DAPI (light blue). Scale bar = 25 µm. (F) Schematic diagram of samples collected for sequencing. (G) PCA analysis of the RNA‐seq of wild‐type (WT) and *Acly* knockout (KO) embryos at the 8‐cell stage. (H) Scatter plots showed the transcriptome changes between WT and KO (left) and siNC and si*Acly* (right) embryos at the 8‐cell stage. Gene expression levels that increased or decreased more than two‐fold are indicated in red or light blue, respectively. FPKM, fragments per kilobase of exon model per million mapped fragments (n = 4, WT and KO; n = 3, siNC and si*Acly*). (I) Heatmap of expression levels for all genes whose expression increased or decreased by more than two‐fold in the KO/WT group (left) and si*Acly*/siNC group (right) (n = 4). (J) The Venn diagram shows overlapping downregulated genes in the KO/WT group and si*Acly*/siNC group. (K) The meta plots showed the global downregulation of H3K27ac signals in the 2 kb window around H3K27acpeaks after knocking down *Acly*. (L) The heatmap of changes in H3K27ac peaks, the definition of differences is more than 1.5‐fold (n = 2). (M) The four‐quadrant diagram shows that the 74% of genes are both downregulated in RNA‐Seq and CUT&Tag (n = 3, RNA‐seq; n = 2, CUT&Tag). (N) The motif analysis of the CUT&Tag of siNC and si*Acly* embryos at the 8‐cell stage. (O) The Venn diagram shows overlapping downregulated genes of RNA‐seq and CUT&Tag after knocking down *Acly*. (P) The biological process GO terms enriched among 599 genes that were both downregulated in RNA‐seq and CUT&Tag after knocking down *Acly*. * *p* < 0.05; ** *p* < 0.01, *** *p* < 0.001. One‐way ANOVA (A); Student's t test (C, E).

Because ACLY is a metabolic enzyme that produces acetyl‐CoA, we evaluated the level of acetyl‐CoA after depletion of *Acly* in early embryos. In *Acly*‐knockout embryos, developmental arrest predominantly occurred at the 8‐cell to morula transition, whereas embryos progressed normally from the 4‐cell to 8‐cell stage (Figure S1O, Supporting Information). By measuring acetyl‐CoA at the 4‐cell stage, we aimed to identify early metabolic perturbations that predict subsequent developmental failure. As expected, siRNA‐mediated depletion of *Acly* (*Acly*‐KD) reduced acetyl‐CoA levels in 4‐cell stage embryos (**Figure** **3**A). Furthermore, embryos derived from mating heterozygous KO mice were immunostained with ACLY to distinguish WT (*Acly* ^+/+^ and *Acly* ^+/–^) from *Acly* KO (*Acly* ^–/–^) embryos. We evaluated the histone acetylation levels at the 8‐cell stage when ACLY was highly expressed. The levels of pan Ac‐H3 decreased during early embryonic development (Figure S3C‐E, Supporting Information), which was likely influenced by acetyl‐CoA levels. We found that *Acly* KO and KD embryos had decreased levels of histone acetylation markers such as H3K27ac (Figure 3B,C, Figure S3G,H, Supporting Information) and H3K9ac (Figure S3J,K, Supporting Information). Notably, although histone acetylation decreased significantly in the si*Acly* embryos, H3K4me3 (Figures S3D and S3F, Supporting Information), H3K9me3 (Figure S3L,O, Supporting Information), H3K27me3 (Figure S3M,O, Supporting Information), and H3K36me3 (Figure S3N,O, Supporting Information) remained unchanged between the groups.

Given the established link between histone acetylation and transcription,^[^ ^56^ ^]^ we examined the nascent RNA levels using EU labeling. Notably, EU signal intensity was significantly decreased in *Acly* KD and KO embryos (Figure 3D,E, Figure S3G, I, Supporting Information). Exogenous acetate administration effectively restored both global histone acetylation and transcriptional activity in ACLY‐depleted embryos (Figure S3G–I, Supporting Information). This indicates that two mechanisms exist for acetyl‐CoA production in early embryos.

To substantiate the impact of ACLY depletion on transcriptional activity, we isolated single embryos harboring the WT (*Acly* ^+/+^ and *Acly* ^+/–^) and *Acly* KO (*Acly* ^–/–^) genotypes at the 8‐cell stage from heterozygous mating mice for RNA‐seq analysis, with four replicates per genotype (Figure 3F). Additionally, 8‐cell embryos with *Acly*‐KD from the zygote stage were collected for RNA‐seq and H3K27ac CUT& Tag (Figure 3F). Principal Component Analysis (PCA) of the RNA‐seq data delineated distinct transcriptomic profiles between WT and ACLY KO embryos (Figure 3G) and between siNC and *siAcly* embryos (Figure S4A, Supporting Information), underscoring significant transcriptome alterations upon ACLY loss. Transcript quantification via Fragments Per Kilobase Million (FPKM) over 1 identified 13 361 and 11 019 genes in WT and *Acly* KO embryos, respectively, and 13 701 and 12 356 genes in siNC and *siAcly* embryos, respectively. Corroborating the EU assay results (Figure 3D,E), ACLY ablation markedly reduced transcription levels, evidenced by the downregulation of 6180 genes and upregulation of 73 genes (|fold change| ≥ 2) in KO embryos (Figure 3H,I, left). Similarly, *Acly‐*KD embryos exhibited downregulation of 5606 genes and upregulation of 202 genes (|fold change| ≥ 2) (Figure 3H,I, right). Notably, 3948 genes were consistently downregulated in both *Acly* KO and *Acly*‐KD embryos, constituting over half of the downregulated genes in the *siAcly* group (3948 out of 5606 genes) (Figure 3J), whereas only four genes were upregulated (data not shown). Gene Ontology (GO) analysis of 240 genes upregulated after *Acly*‐KD or KO showed cell projection organization and cilium assembly (Figure S4B, Supporting Information). GO analysis of the 3, 984 downregulated genes highlighted significant enrichment in biological processes such as chromosome segregation, tricarboxylic acid cycle, histone modification, and spindle organization (Figure S4C, Supporting Information). Collectively, these findings indicate that zygotic loss of ACLY leads to reduced acetyl‐CoA production, diminished histone acetylation, and attenuated transcriptional activity. Interestingly, we found that histone deacetylase genes, such as *Hdac1*, *Hdac2*, and *Hdac3*, were significantly downregulated after knocking down or knocking out *Acly*, indicating the potential role of *Acly* in the regulation of histone modification (Figure 3H,I).

### 2.6 Zygotic *Acly* Deletion Impairs H3K27ac Establishment

Given the established link between H3K27ac and active transcription and the observed reduction in H3K27ac after *Acly* KO or si*Acly* as shown in Figure 3B,C, we conducted low‐input CUT&Tag analyses on H3K27ac in 8‐cell stage embryos with or without knocking down *Acly* (Figure 3F). These results corroborate our immunostaining findings, which revealed a marked reduction in genomic H3K27ac occupancy (Figure 3M). Using macs2, we identified 5301 H3K27ac peaks in 2733 genes. Analysis of the heatmap disclosed that 2327 H3K27ac peaks was diminished post‐*Acly* knocking down, whereas 2632 peaks remained unchanged, only 342 peaks exhibited an upregulation (|fold change in read density| ≥ 1.5) (Figure 3L), highlighting a predominant downregulation of H3K27ac in more than half of the downregulated genes post‐*Acly* deficiency (Figure 3M). To investigate the functional relationship between H3K27ac dynamics and transcriptional regulation systematically, we performed an integrative analysis of chromatin accessibility and gene expression profiles. Based on the RNA‐seq data, we stratified the genes into three distinct categories: upregulated, downregulated, and stable. For each group, we examined the nearest H3K27ac peaks that were predominantly localized in the enhancer regions. Downregulated genes exhibited a significant reduction in H3K27ac signal intensity in *Acly*‐KD embryos compared with controls. The upregulated genes showed increased H3K27ac signals at nearby sites. Stable genes did not show significant H3K27ac changes, confirming their specificity (Figure S4E, Supporting Information). Our analysis revealed that gene expression changes strongly correlated with directional shifts in H3K27ac enhancer activity, supporting ACLY's role in modulating enhancer‐driven transcription via acetyl‐CoA provision.

Moreover, we observed a pronounced decrease in H3K27ac peaks within the promoter regions, augmenting our findings with genomic distributions (Figure S4D,F, Supporting Information). Segmental analysis of both the transcription starting site (TSS) and transcription termination site (TES), focusing on 2 kb bins, confirmed disrupted H3K27ac establishment near promoters, a region typically enriched for H3K27ac (Figure S4F, Supporting Information). Gene Ontology (GO) terms associated with these regions included protein catabolic processes, blastocyst development, and maintenance of cell numbers (Figure S4H, Supporting Information). Similarly, H3K27ac occupancy at the enhancers was compromised (Figure S4G, Supporting Information), with the affected genes predominantly involved in chromatin remodeling, stem cell differentiation, and cell number maintenance (Figure S4I, Supporting Information).

To elucidate the impact of H3K27ac dynamics on early embryonic development, motif analysis of H3K27ac‐enriched downregulated peaks identified several key transcription factors, such as KLF4, GATA3, and TEAD4, which are essential for early embryogenesis (Figure 3N), whereas the upregulated peaks lacked distinct motif signatures (Figure S5A, Supporting Information). Notably, these identified factors were significantly reduced following ACLY suppression (Figure 3H,I), suggesting that ACLY likely facilitates H3K27ac accumulation, which is critical for sustaining vital transcription factor expression during early embryonic stages. Interestingly, the decline in H3K27ac was not attributable to an increase in HDACs (Figure 3H,I), as the levels of *Hdac1*, *Hdac2*, *Hdac3*, and *Hdac4* were concurrently reduced, ruling out HDAC‐mediated effects as a cause of decreased H3K27ac.

Next, we investigated whether the loss of H3K27ac after ACLY loss leads to abnormal transcription. Integrative analysis of RNA‐seq and H3K27ac CUT&Tag data showed that 599 and 610 genes within the H3K27ac‐depleted cohort in *siAcly*‐treated and *Acly* KO embryos, respectively, displayed downregulated expression profiles (RNA‐seq: |fold change in FPKM| ≥ 2; CUT&Tag: |fold change in read density| ≥ 2) (Figure 3O, Figure S5B, Supporting Information). These genes, termed “H3K27ac‐regulated genes” (Figure S5C, Supporting Information), are implicated in processes such as histone modification, cell number maintenance, stem cell population maintenance, regulation of cell division, and blastocyst development (Figure 3P, Figure S5D, Supporting Information), including critical factors like *Klf4*, *Yap1*, *Tfap2c*, and *Pou5f1* (Figure S5E, Supporting Information). Consistent results were obtained using immunofluorescence and RT‐qPCR (Figure S5F–J, Supporting Information). These findings underscore the potential role of ACLY‐driven H3K27ac reduction in mediating widespread transcriptional repression, culminating in the cessation of early embryonic development.

### 2.7 ACLY Displays Nuclear Localization in Human Early Embryos and Exhibits Aberrant Localization in Developmental‐Arrested Embryos

**Figure 4:**
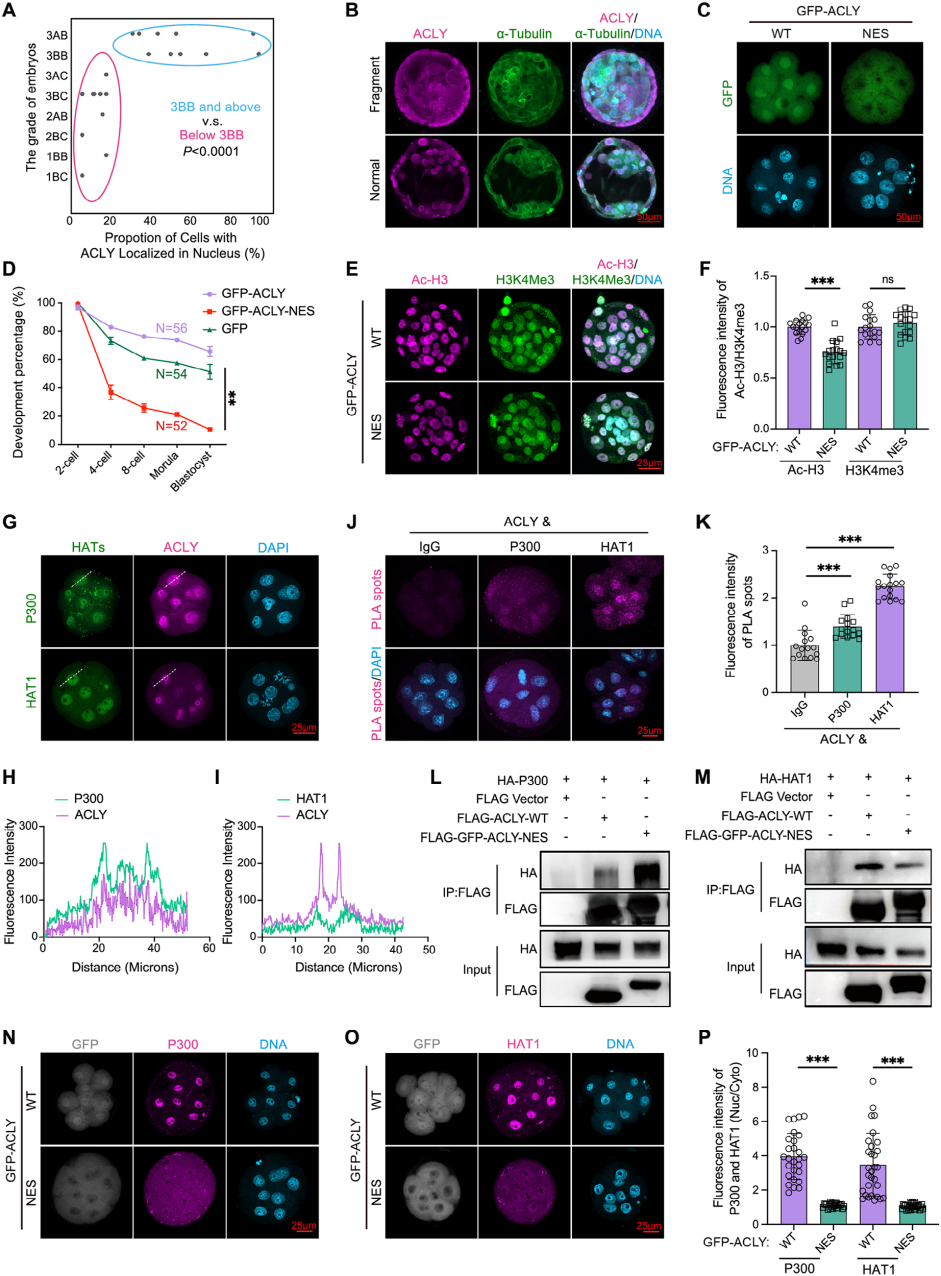
Nuclear localization of ACLY is necessary for early embryo development. (A) Statistical analysis of ACLY localization in blastomeres in donated day 6 human embryos. The proportion of cells with ACLY localized in the nucleus = the number of blastomeres with ACLY localized in the nucleus/total number of blastomeres in the embryo * 100% (n = 24). (B) Representative images of human day 5 normal and fragment embryos stained with anti‐ACLY (violet) and anti‐α‐Tubulin (green) antibodies co‐stained with DAPI (light blue). Scale bar = 50 µm. (C) Representative images of embryos after injection with *Gfp*‐*Acly*‐WT mRNAs or *Gfp*‐*Acly*‐NES mRNAs. GFP was the fluorescent signal fused to ACLY and DAPI were stained for nuclei indication. Scale bar = 50 µm. (D) The development percentage at each stage of embryos developed from zygotes injected with *Gfp*, *Gfp*‐*Acly*‐WT mRNAs, or Flag‐*Gfp*‐*Acly*‐NES mRNAs (n = 56, GFP; n = 52, WT; n = 54, NES; n = 77). (E) Representative images of blastocysts developed from injected zygotes, stained with anti‐Ac‐H3 (violet) antibody and anti‐H3K4me3 (green) antibody (n = 25). Nuclei were stained with DAPI (light blue). Scale bar = 25 µm. (F) Quantitative analysis of the fluorescence intensity of Ac‐H3 and H3K4me3 (n = 17). (G) Representative images of WT embryos stained with anti‐ACLY (violet) antibody and anti‐P300 or anti‐HAT1 antibodies. Nuclei were stained with DAPI (light blue). Scale bar = 25 µm. (H,I) Intensity line profiles of ACLY and P300 (H) / HAT1 (I). (J) Endogenous ACLY could bind with P300 and HAT1 according to Proximity Ligation Assay (PLA) (n = 15). Nuclei were stained with DAPI (light blue). Scale bar = 25 µm. (K) Quantitative analysis of the fluorescence intensity of PLA spots. (L‐M) Exogenous ACLY‐WT/NES could bind with P300 (L) and HAT1 (M) according to the coimmunoprecipitation (Co‐IP) results using NIH3T3. (N‐O) Representative images of embryos stained with anti‐P300 (violet, N) or anti‐HAT1 (violet, O) antibodies after injection with *Gfp*‐*Acly*‐WT mRNAs or *Gfp*‐*Acly*‐NES mRNAs. GFP (gray) was the fluorescent signal fused to ACLY (n = 25). Nuclei were stained with DAPI (light blue). Scale bar = 25 µm. (P) Statistical analysis of the fluorescence intensity ratio of P300 and HAT1 in nucleus and cytoplasm (n = 23, WT; n = 21, NES). Data are presented as mean ± S.D. * *p* < 0.05; ** *p* < 0.01, *** *p* < 0.001. Student's t test (A, D, F, K, and P).

Building on our discovery of the nuclear localization of ACLY in mouse embryos, we evaluated the evolutionary conservation and clinical relevance of ACLY in human embryogenesis. Using 24 morphologically abnormal human embryos discarded from IVF procedures on day 6 post‐fertilization, we performed a standardized quality assessment according to the Gardner grading system.^[^ ^57^ ^]^ Embryos were categorized 13 as of good quality and 11 of poor quality (**Figure** **4**A). IF staining showed that ACLY was predominantly localized in the nuclei of high‐quality embryos, whereas it was distributed more uniformly across the cytoplasm and nucleus in embryos that exhibited developmental delays or fragmentation (Figure 4B). These findings suggested a strong positive correlation between the nuclear localization of ACLY and the quality of early human embryos (Figure 4A,B).

### 2.8 Nuclear Localization and Catalytic Activity of ACLY Is Required for Early Embryo Development

Given the positive correlation between nuclear localization of ACLY and superior‐quality embryos, we determined the functional requirement of this subcellular distribution for embryogenesis. To genetically manipulate ACLY localization, a nuclear export sequence (NES)^[^ ^58^ ^]^ was added to the N‐terminus of the wild‐type GFP‐ACLY plasmid to generate the GFP‐ACLY‐NES plasmid (Figure S6A, Supporting Information). Initially, GFP‐ACLY‐NES was transfected into NIH3T3 and HeLa cells to verify cytoplasmic expression of the resulting protein (Figure S6B, Supporting Information). Subsequently, *Gfp‐Acly* or *Gfp‐Acly‐NES* mRNAs were microinjected into zygotes, demonstrating the predominant nuclear localization of GFP‐ACLY and cytoplasmic distribution of GFP‐ACLY‐NES (Figure 4C). Compared to the *Gfp‐Acly* (WT) microinjection group, the embryo development rate in the *Gfp*‐*Acly*‐NES group decreased progressively across stages, with the final blastocyst formation rate being approximately one‐fifth that of the control group (10.56% versus 65.68%, Figure 4D and S6C, Supporting Information). Further analyses of the effects of GFP‐ACLY‐NES on acetyl‐CoA levels, histone acetylation, and the expression of the embryonic markers CDX2 and NANOG revealed a decrease in acetyl‐CoA levels (Figure S6D, Supporting Information), pan Ac‐H3 (Figure 4E,F), CDX2, and NANOG expression (Figure S6E–G, Supporting Information) post‐*Acly* deletion, without affecting histone methylation markers such as H3K4me3 (Figure 4E,F). This suggests that while endogenous ACLY remains, ectopic GFP‐ACLY‐NES significantly diminishes histone acetylation and crucial developmental markers.

Furthermore, to dissect the mechanism specificity, we generated a catalytically dead mutant of ACLY by mutating histidine at position 760 to alanine (ACLY^H760A^). Microinjection of ACLY^H760A^ mRNAs in wild‐type zygotes recapitulated the ACLY‐NES phenotype, causing significant blastocyst formation failure (Figure S6J,K, Supporting Information), combined with decreased histone acetylation and transcription levels (Figure S6L–O, Supporting Information). These results suggested that both ACLY nuclear localization and enzymatic activity are required for early embryogenesis.

### 2.9 The ACLY‐NES Facilitates the Nuclear Export of HAT1 and P300 Through Protein Interactions

To investigate the spatiotemporal regulation of ACLY‐mediated metabolic‐epigenetic coupling, we pharmacologically ablated HDAC activity using vorinostat (SAHA). This intervention failed to restore blastocyst developmental competence in *Acly* knockdown or ACLY‐NES embryos (Figure S7A–D, Supporting Information). This suggests that competitive sequestration of nuclear acetyl‐CoA pools through HAT mimicry is the underlying mechanism that disrupts histone acetylation. We screened several HATs, including histone acetyltransferase 1 (*Hat1*), lysine acetyltransferase 2A (*Kat2a*), lysine acetyltransferase 2 B (*Kat2b*), CREB‐binding protein (*Cbp*), and E1A binding protein p300 (*P300*). According to the RNA‐seq results from human and mouse embryos,^[^ ^59^ ^]^ *Hat1* emerged as the most abundant HAT (Figure S7E,F, Supporting Information). Colocalization studies revealed that only P300 and HAT1, but not KAT2A, KAT2B, or CBP, were consistently associated with ACLY in the nucleus throughout early embryonic development (Figure 4G–I and Figure S7G, Supporting Information), suggesting a functional interplay. Subsequent proximity ligation assays (PLA) confirmed endogenous ACLY‐HAT1 and ACLY‐P300 interactions in 8‐cell stage embryos (Figure 4J,K). In support of this, co‐immunoprecipitation (co‐IP) assays in NIH3T3 and HEK293T cells demonstrated the binding between wild‐type ACLY and ACLY‐NES with P300 and HAT1 (Figure 4L,M, Figure S7H, Supporting Information).

Notably, ectopic expression of wild‐type ACLY (*Gfp‐Acly* mRNAs) maintained the nuclear localization of HAT1 and P300 in 8‐cell stage embryos (Figure 4N,O), relative to the no‐treatment group (Figure 4G). Interestingly, ACLY‐NES overexpression significantly induced the cytoplasmic export of P300 and HAT1 in 8‐cell embryos (Figure 4N–P). These findings underscore the essential role of ACLY nuclear localization in guiding the nuclear retention of HAT1 and P300, which is critical for successful embryonic development.

### 2.10 HAT1 and P300 Are Required for Histone Acetylation and Early Embryonic Development

**Figure 5:**
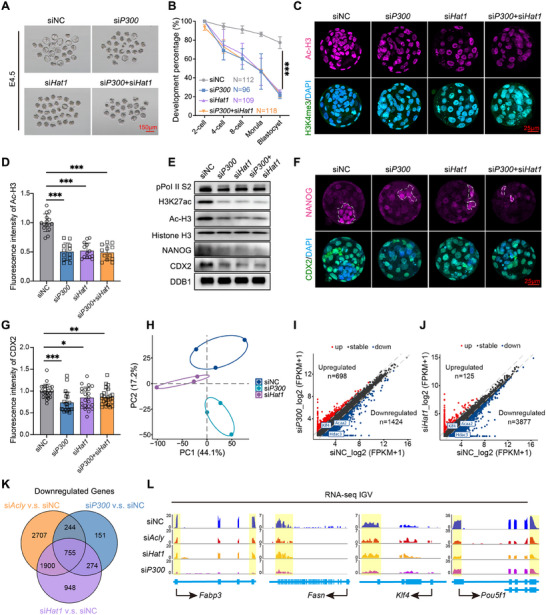
HAT1 and P300 are required for histone acetylation and early embryonic development (A,B) The development process (A) and development percentage (B) at each stage for embryos developed from zygotes injected with 20 µM siNC, si*P300*, si*Hat1*, or both si*P300* and si*Hat1* with 200 ng/µL *Gfp* mRNAs (n = 112, siNC; n = 96, si*P300*; n = 109, si*Hat1*; n = 118, si*P300*+si*Hat1*). Scale bar = 150 µm. (C) Representative images of blastocysts developed from injected zygotes stained with anti‐Ac‐H3 (violet) antibody and anti‐H3K4me3 (green) antibody (n = 23). Nuclei were stained with DAPI (light blue). Scale bar = 25 µm. (D) Quantitative analysis of the fluorescence intensity of H3K4me3 (n = 23). (E) Western blot of 150 E4.5 embryos after different treatments (n = 2). (F) Representative images of blastocysts stained with anti‐NANOG (violet) and anti‐CDX2 (green) antibodies after different treatments (n = 23, siNC; n = 21, siP*300*; n = 22, si*Hat1*; n = 25, si*P300*+si*Hat1*), highlighted areas were inner cell mass. Nuclei were stained with DAPI (light blue). Scale bar = 25 µm. (G) Quantitative analysis of the fluorescence intensity of CDX2 using ImageJ (n = 23, siNC; n = 21, si*P300*; n = 22, si*Hat1*; n = 25, si*P300*+si*Hat1*). (H) PCA analysis of the RNA‐seq of 8‐cell developed from zygotes injected with 20 µM siNC, si*P300*, si*Hat1*, or both si*P300* and si*Hat1* with 200 ng/µL *Gfp* mRNA. (I‐J) Scatter plots showing the transcriptional differences between siNC and si*P300* (I) or siNC and si*Hat1* (J) at the 8‐cell stage. Genes with expression levels that increased or decreased by more than twofold are indicated in red and light blue, respectively (n = 3). (K) Venn diagram showing the overlap of downregulated genes among the three groups. Downregulated and upregulated genes are defined as those with expression that increased or decreased by more than twofold. FPKM, fragments per kilobase of exon model per million mapped fragments (n = 4). (L) Genome browser view showing the FPKM of *Fabp3*, *Fasn*, *Klf4*, and *Pou5f1* in control (siNC), knocking down *Acly* (si*Acly*), *P300* (si*P300*), or *Hat1* (si*Hat1*) 8‐cell embryos. Data are presented as mean ± S.D. *p < 0.05; **p < 0.01, ***p < 0.001. One‐way ANOVA (B, D, and G).

Given the role of ACLY in orchestrating the nuclear localization of HAT1 and P300, we investigated the functional contributions of these histone acetyltransferases to early embryonic development. P300 and HAT1 were depleted by siRNA‐mediated knockdown. Consistent with the *Acly*‐KD results, both *P300*‐KD and *Hat1*‐KD embryos exhibited progressive developmental retardation (**Figure** **5**A,B). The final blastocyst formation rate was approximately 1/3 of that in the siNC group (Figure 5A,B). The RT‐qPCR and immunofluorescence results showed that *P300* and *Hat1* were successfully depleted (Figure S7I‐K). Surprisingly, si*P300* or si*Hat1* alone caused significant embryo arrest, and knocking down both *P300* and *Hat1* did not synergistically enhance the phenotype (Figure 5A,B), suggesting functional compensation between these HATs. Histone acetylation (Figure 5C–E), transcription levels (Figure 5E), and cell lineage genes (CDX2 and NANOG) (Figure 5E–G) also decreased after microinjection of si*P300* or si*Hat1* siRNAs, consistent with the phenotype of si*Acly*.

In addition, single‐embryo RNA‐seq was performed on embryos in the *P300*‐knockdown (si*P300*) and *Hat1*‐knockdown (si*Hat1*) groups. PCA cluster analysis revealed different distributions among widetype, *P300*‐knockdown, and *Hat1*‐knockdown embryos (Figure 5H). A total of 13701, 13314, and 11893 genes were identified in the siNC, si*P300*, and si*Hat1* embryos, respectively. There were more downregulated genes than upregulated genes (*P300*‐knockdown: 1424 downregulated genes, 698 upregulated genes; *Hat1*‐knockdown: 3877 downregulated genes, 125 upregulated genes, |fold change| ≥ 2) (Figure 5I,J). Notably, 70.15% of the downregulated genes in the si*P300* group and 68.48% of the downregulated genes in the si*Hat1* group were also downregulated in the si*Acly* group (Figure 5K). There were 775 overlapping down‐regulated genes in all three groups (Figure 5K). Further analysis of these 755 overlapping downregulated genes revealed several fatty acid metabolism and cell lineage differentiation‐related genes, such as *Fabp3*, *Fasn*, *Klf4*, and *Pou5f1* (Figure 5L). These findings imply that P300 and HAT1 play partial roles in mediating histone acetylation and transcriptional promotion of ACLY.

### 2.11 AKT Pathway Facilitates ACLY Phosphorylation and the Nuclear Localization of ACLY and P300/HAT1

**Figure 6:**
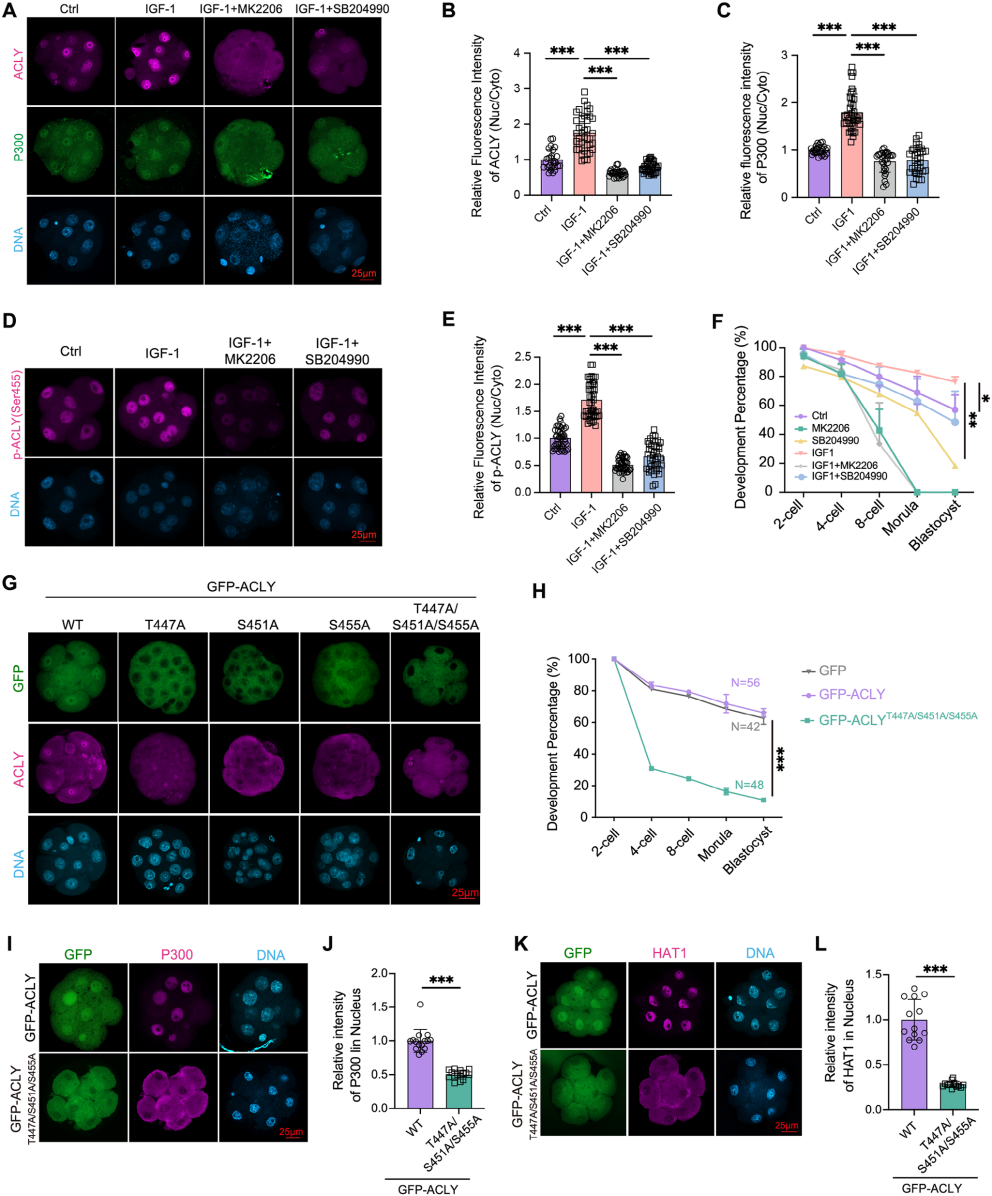
AKT pathway activation facilitates ACLY phosphorylation and the nuclear localization of ACLY and P300/HAT1 (A) Representative images of embryos treated with 10 µM DMSO, 10 ug/ml IGF‐1, both IGF‐1 and MK2206, or both IGF‐1 and 20 µM SB204990 from the zygote stage stained with anti‐ACLY (violet) and P300 (green) antibodies. Nuclei were stained with DAPI (light blue). Scale bar = 25 µm. (B‐C) Quantitative analysis of the fluorescence intensity ratio of ACLY (C) and P300 (D) in nucleus and cytoplasm using ImageJ (n = 26, ctrl; n = 40, IGF‐1; n = 29, IGF‐1+MK2206; n = 34, IGF‐1+SB204990). (D) Representative images of embryos stained with anti‐p‐ACLY (Ser455) (violet) antibody after different treatments. Nuclei were stained with DAPI (light blue). Scale bar = 25 µm. (E) Quantitative analysis of the fluorescence intensity of ratio of p‐ACLY (Ser455) in nucleus and cytoplasm (n = 44, ctrl; n = 47, IGF‐1; n = 43, IGF‐1+MK2206; n = 43; IGF‐1+SB204990). (F) The development percentage at each stage of embryos after different treatments (n = 80, ctrl; n = 64, MK2206; n = 93, SB204990; n = 80, IGF‐1; n = 57, IGF‐1+MK2206; n = 63, IGF‐1+SB204990). (G) Representative images of embryos stained with anti‐ACLY (violet) antibody, after injecting with a single T447A, S451A, or S455A mutation *Acly* mRNA. GFP (green) was the fluorescent signal fused to ACLY and nuclei were stained with DAPI (light blue). Scale bar = 25 µm. (H) The development percentage at each stage of the embryos, which were developed from zygotes injected with *Gfp*, *Gfp*‐*Acly*‐WT, or *Gfp*‐*Acly* ^T447/S45A/S455A^ mRNA (n = 3). (I–K) Representative images of embryos stained with anti‐P300 (I) or anti‐HAT1 (K) (violet) antibodies, which were developed from injected zygotes with *Gfp*‐*Acly*‐WT or *Gfp*‐*Acly* ^T447/S45A/S455A^ mRNAs. GFP (green) was the fluorescent signal fused to ACLY. Nuclei were stained with DAPI (light blue). Scale bar = 25 µm. (J–L) Quantitative analysis of the fluorescence intensity ration of P300 (J) and HAT1 (L) in nucleus and cytoplasm using ImageJ (P300: n = 15, *Gfp*‐*Acly*‐WT; n = 15, *Gfp*‐*Acly* ^T447/S45A/S455A^; HAT1: n = 13, *Gfp*‐*Acly*‐WT, n = 13, *Gfp*‐*Acly* ^T447/S45A/S455A^). Data are presented as mean ± S.D. *p < 0.05; **p < 0.01; ***p < 0.001. Student's t test (J and L). One‐way ANOVA (B, C, E, F, and H).

Emerging evidence indicates that AKT‐mediated phosphorylation of ACLY at Ser455 facilitates nuclear acetyl‐CoA production, which is essential for histone acetylation and BRCA1 recruitment during DNA damage repair.^[^ ^45^ ^]^ As IGF‐1 is an activator of the AKT signaling pathway, we found that the concentration of IGF‐1 exhibited dynamic changes in both the fallopian tube and uterine fluids, with a notable peak in IGF‐1 levels observed in the fallopian tube fluid at the 8‐cell stage (Figure S8A). This temporal correlation prompted us to investigate whether AKT‐dependent ACLY phosphorylation occurs during normal embryogenesis. To test this hypothesis, we used IGF‐1 and MK2206, which are known to activate and inhibit the AKT pathway, respectively.^[^ ^60^, ^61^ ^]^ Embryos were cultured in KSOM medium supplemented with either DMSO or 2 µM MK2206, an AKT inhibitor. Compared with 8‐cell stage embryos in the control group, MK2206 treatment for 12 h in 4‐cell stage embryos markedly induced a uniform cellular distribution of ACLY in 8‐cell stage embryos (Figure S8B,C. Supporting Information), and decreased the p‐ACLY (S455) level in 8‐cell embryos (Figure S8D,E, Supporting Information). Conversely, addition of IGF‐1 (10 µg/mL) in culture medium significantly stimulates the nuclear localization of p‐AKT (S473) (Figure S8F,G, Supporting Information), the nuclear localization of ACLY (**Figure** **6**A,B) and P300 (Figure 6A,C), and it also increased the p‐ACLY (S455) level in nucleus (Figure 6D,E) and increased the H3K27ac level (Figure S8F–H, Supporting Information) in 8‐cell embryos. In contrast, treatment with MK2206 impeded the IGF‐1‐induced increase in p‐AKT (S473) (Figure S8F,G, Supporting Information) and p‐ACLY (S455) (Figure 6D,E) and inhibited the nuclear localization of ACLY and P300 in 8‐cell embryos (Figure 6A–C). Similar to *Acly*‐KD or *P300*‐KD, AKT or ACLY inhibition blocked the increase in H3K27ac levels even with IGF‐1 (Figure S8F,H, Supporting Information). Notably, the ACLY inhibitor (SB204990) induced the cytoplasmic localization of P300 (Figure 6A,C, Supporting Information).

Considering that changes in histone acetylation levels indicate a disruption in the balance between HDACs and HATs, we examined the response of HDACs to IGF‐1. Our results revealed that IGF‐1 supplementation did not significantly alter the total HDAC1/2/3 protein levels or p‐HDAC2 (Ser394) (p‐HDAC2, an activated form) (Figure S8I–M, Supporting Information). This implies that IGF‐1 the role in enhancing histone acetylation (e.g., H3K27ac) is likely mediated through mechanisms independent of HDAC suppression, such as modulation of HAT activity. These results suggest that the AKT pathway facilitates ACLY phosphorylation and the nuclear localization of ACLY and P300/HAT1.

To delineate the mechanistic dependence of IGF‐1′s pro‐developmental effects on ACLY, combinatorial pharmacological interventions were performed. Consistent with previous studies,^[^ ^62^ ^]^ IGF‐1 augmented blastocyst formation (Figure 6F, Figure S8N,O, Supporting Information) and increased the number of blastomeres in mouse embryos (Figure S8N,O, Supporting Information). Conversely, the inhibition of AKT by MK2206 and ACLY by SB204990 curtailed blastocyst formation (Figure 6F) and decreased blastomere formation (Figure S8N,O, Supporting Information). These data establish that phosphorylated ACLY is a critical metabolic effector downstream of AKT signaling, functionally coupling with IGF‐1 stimulation to enhance embryonic developmental competence.

### 2.12 ACLY Phosphorylation Is Responsible for Its Nuclear Localization, and Recruits the Nuclear Localization of P300 and HAT1 in Early Embryos

We systematically investigated the AKT‐mediated phosphorylation of ACLY in early embryos to elucidate the mechanistic link between ACLY phosphorylation and HATs recruitment. Building on prior reports of AKT‐dependent phosphorylation at Ser455 during the DNA damage response,^[^ ^45^ ^]^ we employed bioinformatic prediction (https://www.phosphosite.org/) to identify two additional putative AKT phosphorylation sites: Thr447 and Ser451 (Figure S9A, Supporting Information). Site‐specific mutant constructs (T447A, S455A, and S451A) were generated to assess their effects on ACLY's cellular localization of ACLY (Figure S9B, Supporting Information). Notably, mutations at T447A and S455A significantly affected ACLY's nuclear localization of ACLY, whereas the S451A mutation had a minimal impact (Figure S9C, Supporting Information). To further explore the roles of these phosphorylation sites, a triple mutant (T447A/S451A/S455A) was engineered. Following transfection into HeLa cells, a significant reduction in the overall ACLY phosphorylation was observed by immunoblotting with an anti‐FLAG antibody (Figure S9D,E, Supporting Information). These results indicate that T447, S451, and S455 may be phosphorylation sites.

Next, we studied the role of these sites in nuclear localization of GFP‐ACLY in early embryos. Each site mutation and triple mutation were tested in early embryos by microinjecting *Gfp‐Acly* mRNAs harboring these mutations into zygotes. Mutation of these three sites in GFP‐ACLY^T447/S451/S455A^, led to pronounced cytoplasmic localization of exogenous ACLY (Figure 6G), slowed embryonic development, and decreased blastocyst formation rates compared with those in embryos injected with wild‐type *Gfp‐Acly* mRNAs (Figure 6H). Similar to GFP‐ACLY‐NES, the triple mutant GFP‐ACLY^T447/S451/S455A^ induced cytoplasmic localization of HAT1 and P300 (Figure 6I–L), correlating with reduced Ac‐H3 (Figure S9F,G, Supporting Information) and abnormal lineage differentiation in blastocysts (Figure S9H,I, Supporting Information). Taken together, these results demonstrate the pivotal role of the S455, T447, and S451 phosphorylation sites in regulating ACLY's nuclear localization of ACLY and highlight their importance in recruiting the nuclear localization of HATs for histone acetylation.

### 2.13 Maternal ACLY Is Dispensable For Oogenesis and Embryogenesis Due to the Compensatory Upregulation of ACSS2 in Oocyte

Given ACLY's high expression in oocytes, we investigated whether maternal ACLY was essential for embryogenesis. Genotyping, RT‒qPCR, and western blot (WB) verified that *Acly* was efficiently knocked out in oocytes at the DNA, mRNA, and protein levels (Figure S1N and S10A–D, Supporting Information). First, we analyzed the average litter size. The breeding assay showed that *Acly* cKO mice were fertile, and comparable litter sizes were obtained from wild‐type (*Acly^flox/flox^*, WT) and *Acly* cKO mice after mating with wild‐type adult male mice for over six months (Figure S10E, Supporting Information). Next, we assessed the follicular, meiotic, and in vivo early embryonic development in *Acly* cKO mice. As shown in Figure S10F (Supporting Information), *Acly* cKO mice had grossly normal ovarian histology compared to WT mice at six months of age. Both WT and *Acly* cKO mice ovulated mature (MII phase) oocytes with normal spindle morphology (Figure S10G–J, Supporting Information). The WT and *Acly* cKO female mice were mated with wild‐type male mice, and the resulting zygotes were cultured in vitro. Normal development of early embryos at each stage was observed in both groups (Figure S10K,L, Supporting Information). Based on these results, we concluded that maternal *Acly* deficiency in oocytes is dispensable for oogenesis, meiotic maturation, and embryonic development.

**Figure 7:**
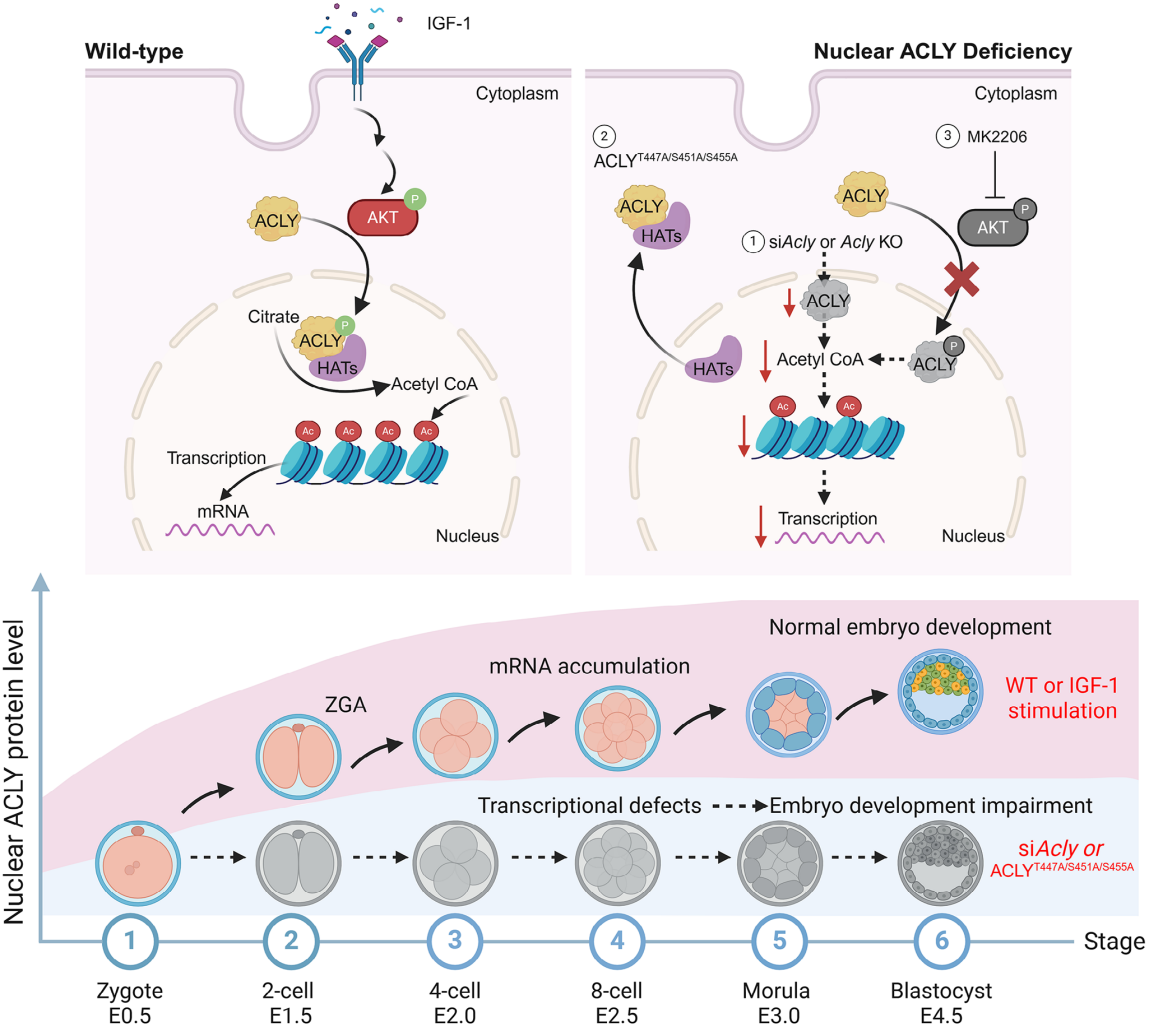
The nuclear localization of ACLY guards early embryo development through Recruiting P300 and HAT1 to promote histone acetylation and mRNA Transcription. From zygote to blastocyst, the normal function of ACLY is crucial for embryonic development. In wild‐type embryos, IGF‐1 stimulates AKT, which subsequently phosphorylates ACLY, enhancing its nuclear localization and its ability to recruit histone acetyltransferases (HATs) such as P300 and HAT1. This recruitment increases Acetyl‐CoA production and histone acetylation within the nucleus, crucial for robust transcriptional activity. Conversely, siRNA‐mediated knockdown or genetic knockout of *Acly* diminishes its activity, leading to reduced acetyl‐CoA production and attenuated transcriptional activity. This reduction can also be achieved by inhibiting AKT with MK2206 or by inactivating ACLY phosphorylation at specific sites (ACLY^S447A/S451A/S455A^), further exacerbating the consequences of nuclear ACLY deficiency. Deficient nuclear ACLY induces cytoplasmic localization of HATs, resulting in decreased histone acetylation, reduced transcriptional activity, and subsequent early embryonic arrest.

Building on these findings, we explored whether ACSS2 compensates for ACLY deficiency in oocytes. As anticipated, ACLY was undetectable in *Acly* cKO oocytes (Figure S10B,C, Supporting Information), whereas ACSS2 protein levels were markedly elevated compared to those in wild‐type (WT) growing oocytes (Figures S10C,D and S10M,N, Supporting Information). In WT‐growing oocytes, siRNA‐mediated *Acss2* depletion (si*Acss2*) triggered significant ACLY upregulation (Figure S10O,P, Supporting Information). This complementary increase in ACSS2 may contribute to comparable levels of H3K27ac in *Acly* cKO growing oocytes (Figure S10Q,R, Supporting Information).

Both ACLY and ACSS2 were localized in the nucleus of growing and fully grown oocytes (Figure S11A, Supporting Information). Furthermore, to test for functional redundancy during oocyte maturation, we performed single or double knockdown of *Acly* and *Acss2* in fully grown GV oocytes. A single knockdown of *Acss2* impaired GVBD progression but did not affect MII maturation rates in oocytes that successfully underwent GVBD (Figure S11B–D, Supporting Information). *Acly* knockdown alone did not affect GVBD or MII rates (Figure S11B–D, Supporting Information), which was consistent with *Acly* cKO phenotypes. The knockdown of *Acly* and *Acss2* induced significant reductions in GVBD and MII rates (Figure S11B–D, Supporting Information) and disrupted spindle assembly (Figure S11E,F, Supporting Information). This indicates that ACSS2 likely plays a more vital role in oocyte maturation than ACLY does. In summary, ACLY and ACSS2 exhibit partial redundancy during oogenesis and oocyte meiotic maturation, and ACSS2 is more important than ACLY in the initiation and resumption of meiotic maturation.

## 3 Discussion

Recent advances in developmental biology have uncovered a complex interplay between cellular metabolism and epigenetic regulation during early embryogenesis.^[^ ^12^, ^63^ ^]^ Our study identified ACLY's unique nuclear localization in early embryos. ACLY serves as a central metabolic orchestrator that bridges nutrient sensing with chromatin remodeling by spatially regulating acetyl‐CoA synthesis. ACLY compartmentally catalyzes acetyl‐CoA production and simultaneously recruits P300 and HAT1 to the nucleus for nearby histone acetylation, bypassing the limitations of passive metabolite diffusion(Figure 7). Although mitochondrial enzymes such as pyruvate dehydrogenase alpha 1 (PDH1) facilitate ZGA through nuclear acetyl‐CoA generation,^[^ ^64^, ^65^ ^]^ we demonstrated that ACLY governs post‐ZGA (8‐cell to morula transition) epigenetic programming through phosphorylation‐dependent nuclear translocation. Our findings not only provide further evidence for the spatiotemporal metabolic control of epigenetic modification and gene expression during early embryogenesis, but also offer a metabolic rationale for the environmental stimulation (e.g., IGF‐1)‐mediated AKT signaling pathway, histone acetylation, and embryo development promotion.

The inconsistent consequences of ACLY depletion in oocytes and early embryos revealed a fundamental divergence between oocyte and embryonic metabolic regulation, while highlighting the developmental vulnerabilities of early embryos. Maternal ACLY ablation in oocytes is associated with compensatory ACSS2 upregulation, a response potentially mediated by granulosa cell signaling and acetate metabolic exchange within the follicular niche.^[^ ^66^, ^67^, ^68^, ^69^ ^]^ This aligns with somatic systems, in which ACLY‐ACSS2 functional redundancy enables metabolic compensation, as evidenced in macrophages, adipocytes, embryonic fibroblasts, and several tumor cells.^[^ ^70^, ^71^, ^72^, ^73^ ^]^ Strikingly, zygotic ACLY deletion caused irreversible developmental arrest at the 8‐cell to morula transition, whereas acetate supplementation partially rescued acetyl‐CoA and epigenetic levels through ACSS2‐mediated acetyl‐CoA production. Failure to restore embryonic development underscores the attenuated compensatory network and the unique role of nuclear ACLY during early embryonic development. Our study revealed a more important role for ACSS2 in oogenesis and oocyte maturation, suggesting a niche‐dependent regulation of metabolic plasticity. It seems that ACLY is more critical for early embryo development, probably because early embryo development is relatively auto‐organized, depending on maternal substances (mRNA or proteins).^[^ ^74^, ^75^, ^76^, ^77^ ^]^ Overall, the results of this study deepen our understanding of the relationship between ACLY and ACSS2 during oogenesis and early embryonic development.

During mammalian MZT, embryonic genome activation unfolds through transcriptionally distinct waves.^[^ ^74^, ^75^, ^78^ ^]^ In mice, ZGA is initiated at the 2‐cell stage, followed by MGA at the 8‐cell stage, which is a critical phase for trophectoderm and inner cell mass lineage specification.^[^ ^79^, ^80^, ^81^ ^]^ Multiomics studies have revealed metabolic‐epigenetic crosstalk during these stages; TCA metabolites regulate ZGA via H3K27ac dynamics, whereas NAD^+^ mediates H3K27ac removal to exit minor ZGA.^[^ ^9^, ^31^, ^50^, ^82^ ^]^ Epigenetic marks like H3K4me3, H3K27me3, and H3K27ac coordinate transcriptional bursting and developmental competence,^[^ ^20^, ^83^, ^84^, ^85^, ^86^, ^87^ ^]^ with L‐2‐hydroxyglutarate (L‐2‐HG)‐driven H3K4me3 defects disrupting blastocyst formation.^[^ ^16^ ^]^ Despite these advances, key knowledge gaps persist: (1) the enzymatic drivers coordinating metabolic inputs with histone modification dynamics remain undefined, and (2) the mechanistic links between metabolic flux and genome‐wide epigenetic reprogramming post‐ZGA are poorly characterized. Our study addresses these gaps by identifying ACLY as a metabolic‐epigenetic nexus that governs early embryogenesis. ACLY depletion disrupts global histone acetylation, with H3K27ac exhibiting the most pronounced reduction at pluripotency loci. Spatial profiling revealed collapsed H3K27ac occupancy at the promoters or enhancers of downregulated genes, including critical lineage specifiers, which correlated with transcriptional silencing and developmental arrest at the 4‐cell stage. These findings suggest that ACLY is not merely an acetyl‐CoA supplier but also a transcriptional co‐regulator that licenses genome activation through metabolic‐epigenetic integration.

Generally, ACLY is localized in the cytoplasm of multiple cell types.^[^ ^40^, ^88^, ^89^ ^]^ A previous study found that nuclear localization of ACLY allows the rapid production of acetyl‐CoA in the nucleus in response to DNA damage.^[^ ^45^ ^]^ Overexpression of nuclear‐excluded ACLY (NES) mutants or catalytically inactive ACLY (*Acly* ^H760A^) mutants in wild‐type zygotes recapitulated blastocyst formation defects and histone hypoacetylation, indicating that both mutants likely acted as dominant‐negative variants by competing with endogenous ACLY. This functional convergence revealed the essential requirements for ACLY enzyme activity and nuclear localization to sustain developmental competence. Through the ACLY‐NES model, we pioneer the discovery that metabolic enzyme compartmentalization actively governs HAT localization—a paradigm shift transcending the classical “metabolite‐centric” view toward an integrated epigenetic regulatory framework incorporating both metabolite flux and subcellular compartmentalization. Our findings established that ACLY is a spatial conductor that physically anchors HATs to the chromatin by coupling nuclear acetyl‐CoA synthesis with site‐specific histone modifications. This redefines metabolic‐epigenetic crosstalk as a geometrically constrained process in which enzyme positioning (not merely metabolite abundance) dictates cell fate transitions.

Mechanistically, *Acly* knockdown not only affected histone acetylation but also triggered the coordinated downregulation of HDAC1/2/3 at both the mRNA and protein levels. At the same time, the HDAC inhibitor SAHA failed to rescue the developmental defects. Furthermore, cell proliferation requires enhanced histone acetylation activity because cells lacking P300 activity display proliferation defects.^[^ ^90^, ^91^ ^]^ Consistently, IGF‐1 treatment induces AKT‐ACLY‐mediated HATs nuclear localization, H3K27ac increase, and embryo development, revealing a broken balance between HATs and HDACs.^[^ ^92^, ^93^ ^]^ Overall, these results indicate that histone acetylation dynamics during blastocyst formation are governed by the competing regulation of HDAC attenuation and HAT potentiation, and that a low HAT‐HDAC balance is detrimental to embryo development. In our study, AKT‐mediated phosphorylation (T447/S451/S455) emerged as a key regulator of ACLY nuclear translocation, aligns with somatic systems after DNA damage or obesity‐related factors (estradiol, insulin and leptin).^[^ ^45^, ^94^, ^95^, ^96^ ^]^ Phosphorylated ACLY recruits HAT1 and P300 through direct physical interactions, forming nuclear metabolons that sustain H3K27ac at pluripotency loci (such as *Pou5f1*). The temporal concordance between ACLY nuclear enrichment and elevated IGF‐1 levels in fallopian tube fluid at the 8‐cell stage suggests systemic endocrine‐metabolic coordination during developmental milestones.

Three key limitations contextualize our findings: 1) unresolved citrate/isocitrate flux dynamics requiring isotope‐assisted flux analysis, 2) technical constraints in embryoscale acetyl‐CoA measurements, and 3) an undefined structural basis of ACLY‐P300 or HAT1 interactions. Future studies that integrate single‐cell metabolomics, nuclear‐localized acetyl‐CoA biosensors, and cryoelectron microscopy will deepen our understanding of early embryonic development.

## 4 Experimental Section

### Clinical samples

Day 5 embryos with normal developmental potential were donated by a patient who had delivered two live births and elected not to pursue further pregnancies. Day 6 embryos were graded according to the Gardner grading system.^[^ ^57^ ^]^ All experiments were approved by the ethics committee of Sir Run Run Shaw Hospital affiliated to Zhejiang University, under certification number 20210319–32. All patients provided written informed consent to the collection of surplus day 6 embryos.

### Animals

The mouse care and animal experimental procedures in this study were conducted in accordance with the Animal Research Committee guidelines of Zhejiang University. All mice were housed in the Sir Run Run Shaw Hospital animal facility affiliated with Zhejiang University with a 14/10 h light/dark cycle under specific pathogen‐free animal facility.

All wild‐type (WT) C57BL/6 mice used in this study were obtained from the Shanghai SLAC Experimental Animal Co., Ltd., China. The *Gdf9‐cre* and *Ddx4‐Rfp* mice have been described previously. *Acly^flox/flox^* mice with a deletion from exon 3 to exon 6 (Figure S2A, Supporting Information) were obtained from GemPharmatech (China, T018441). The *Acly^flox/flox^* mice were crossed with *Gdf9‐cre* mice to obtain female *Acly^flox/flox^; Gdf9‐cre* mice, which were then crossed with wild‐type male mice to obtain *Acly* heterozygotes (*Acly* ^+/–^). The genotyping primers are listed in Table S2 (Supporting Information). The results of the genotyping gel are shown in Figure S1I (Supporting Information).

### Cell lines

Human embryonic kidney cells (HEK293T) and human cervical carcinoma cells (HeLa) were cultured in DMEM (Gibco, USA, C11995500BT) supplemented with 10% fetal bovine serum (FBS, CellMax, China, SA101.01) and 1% penicillin‒streptomycin solution (Gibco, USA, 15140122) at 37 °C with humidified 5% CO2.

### Collection of Oocytes and Early Embryos

To obtain oocytes or early embryos, 3‐ to 4‐week‐old C57BL/6 female mice were intraperitoneally injected with 5.0 IU pregnant mare serum gonadotrophin (PMSG; Sansheng, China, 110254564), followed 48 h later by injection of 5.0 IU of human chorionic gonadotropin (hCG; Sansheng, China, 110251282). Fully grown oocytes (>70 µm) were collected from the ovaries of C57BL/6 female mice 48 h after the injection of PMSG. Early embryos were collected from female mice mated with C57BL/6 wild‐type male mice; successful mating was confirmed by the presence of a vaginal plug. Early embryos were collected at the following time points after hCG stimulation: PN3 zygote embryos (20–22 h), early 2‐cell embryos (30 h), late 2‐cell embryos (46–48 h), 4‐cell embryos (54–56 h), 8‐cell embryos (68–70 h), morula (76–78 h) and blastocysts (96 h). Zygotes were collected from the ampullae of oviducts and released from cumulus cells in M2 medium containing 100 µg/mL hyaluronidase (Sigma, USA, H1115000). Embryos at the 2‐cell, 4‐cell, 8‐cell, and morula stages were collected from the oviducts, while blastocysts and post‐implantation embryos were obtained from the uterus. For embryonic day 6.5 (E6.5) embryo acquisition, the uterus was fixed with 4% paraformaldehyde (PFA, Solarbio, China, P1110) and offspring tissue was obtained from paraffin sections with a glass needle under a stereoscope.

### In Vitro Culture of Mouse Embryos

Zygotes were cultured in a 40 µL drop of preequilibrated KSOM medium (Easy check, China, M1430) covered by mineral oil (Sigma, USA, M5310) at 37 °C in a humidified 5% CO_2_ environment. To inhibit the function of ACLY, 20 µM DMSO or SB204990 (MCE, USA, HY‐16450) was added to KSOM. IGF‐1 (MCE, USA, HY‐P7018) was added to activate the AKT pathway. SAHA (MCE, USA, HY‐10221) was added to inhibit HDACs.

### Evaluation of Transcription by EU Staining

Transcriptional capacity was tested using the Click‐iT RNA Alexa Fluor 488 Synthesis Assay Kit (Thermo Fisher, USA, C10329). Embryos were incubated in KSOM medium with 100 µM 5‐ethynyluridine (EU) for another 1 h before fixation with 4% PFA. The subsequent steps were performed according to the manufacturer's protocol.

### Evaluation of Acetyl‐CoA Level

Acetyl‐CoA levels were tested using the Acetyl‐CoA Assay Kit (Sigma, USA, MAK039). For each sample, 500 4‐cell embryos were collected after removing the zona pellucida with Tyrode's solution (Sigma, USA, T1788) and then stored at −80 °C until the desired number of embryos was reached. The subsequent steps were performed according to the manufacturer's protocol.

### Evaluation of IGF‐1 Level in Fallopian Tubes and Uterus

IGF‐1 levels were measured using the Mouse/Rat IGF‐1 ELISA Kit (Proteintech, USA, KE10032). At each time point, the fallopian tubes and uterus from three mice were aseptically dissected and placed in separate sterile tubes. The reproductive tracts were flushed with 100 µL of KSOM medium using a blunt‐end needle, and the collected medium were centrifuged at 1000 × g for 15 min at 4 °C. The supernatant was aliquoted for subsequent analysis, and the remaining steps followed the manufacturer's protocol.

### Plasmid Construction

Full‐length human *ACLY* and *HAT1* cDNA were obtained from the human Ultimate ORF clone library (Thermo Fisher, USA, HORF01). After sequencing, FLAG or HA‐labeled expression plasmids were generated by using LR clonase (Thermo Fisher, USA, 11 791 019), according to the manufacturer's instructions. The HA‐P300 plasmid was a gift from Professor Cao Liping. The nuclear export plasmid was constructed by adding a nuclear export sequence (NES, 5′‐AGT CTG GCA GCT GAG TTC CGA CAC CTG CAA CTG AAG GAA‐3′) or nuclear localization sequence (NLS, 5′‐CCA AAG AAG AAG CGA AAG ATG‐3′) to the N‐terminus of the wild‐type (WT) ACLY plasmid. NES/NLS forward and reverse full‐length primers, with sticky ends generated by *EcoR I* (Thermo Fisher, USA, FD0274) and *EcoR V* (Thermo Fisher, USA, ER0301), were used to synthesize double‐stranded DNA in vitro with an annealing buffer (Beyotime, China, D0251). Then, the double‐stranded DNA with sticky ends was inserted into the wild‐type ACLY plasmid by enzyme ligation. All plasmids were confirmed by Sanger sequencing before use.

### Plasmid Transfection

Plasmid transfection was conducted with Lipofectamine 3000 Transfection Reagent (Thermo Fisher, USA, L3000015) according to the manufacturer's protocol once cells reached 50–60% confluence.

### Coimmunoprecipitation (co‐IP)

Cells were transiently transfected for 48 h and washed twice with cold PBS (Genom, China, CNM20012‐2). Next, the cells were collected with IP lysis buffer (Beyotime, China, P0013) and lysed on ice for 30 min. After centrifugation at 15 000 rpm for 15 min at 4 °C, 100 µL of supernatant was collected as input. The residual supernatant was incubated overnight at 4 °C with anti‐Flag magnetic beads (Sigma, USA, M8823). The beads were collected after three washes with IP lysis buffer using a magnetic frame, and the coimmunoprecipitated proteins were released by adding 1×Laemmli protein sample buffer (BIO‐RAD, USA, 1610 747).

### Proximity Ligation Assay (PLA)

The detection of endogenous protein interactions used Duolink In Situ PLA assay (Sigma, USA, DUO92101). Embryos were collected at corresponding time point and then fixed in 4% PFA in PBS at room temperature for 30 min. The subsequent steps were performed according to the manufacturer's protocol.

### Western Blot Analysis

Embryos were lysed directly in 1× Laemmli sample buffer, and cells were lysed in RIPA lysis buffer (Solarbio, China, R0010) followed by the addition of 4× Laemmli sample buffer (BIO‐RAD, USA, 1610747). The resulting protein samples were denatured at 98 °C for 5 min and subjected to separation by sodium dodecyl sulfate‒polyacrylamide gel electrophoresis, followed by gel transfer to polyvinylidene difluoride (PVDF) membranes (Millipore, USA). Next, the membranes were blocked in 5% skimmed milk for 1 h and then incubated at 4 °C overnight with primary antibodies. Membranes were then incubated at room temperature with secondary antibodies (CST, USA, 7074 and 7076) for 1 h and washed with TBST buffer three times. Finally, the membranes were visualized using an enhanced exposure solution (Millipore, USA, P90720) and a chemical imaging system (BIO‐RAD, USA, 10044275).

### In Vitro Transcription

The wild‐type and mutant FLAG‐ACLY plasmids were digested using Hind III. Then, 5′‐capped mRNAs were transcribed using the Sp6 mMESSAGE mMACHINE Kit (Thermo Fisher, USA, AM1340), followed by poly(A) tail addition using a Poly(A) Tailing Kit (Thermo Fisher, USA, AM1350). The synthesized poly(A) mRNAs were recovered with lithium chloride precipitation overnight at −20 °C, washed with 70% ethanol, and dissolved in nuclease‐free water.

### Microinjection of Embryos with siRNAs or mRNAs

PN3 murine zygotes were microinjected with siRNA or reporter mRNA. *Gfp* or *mCherry* mRNA was included as a marker to ensure successful microinjection. All siRNAs were purchased from Ribo Biotechnology Co., Ltd. Microinjections were performed using a Narishige micromanipulator. Approximately 10 pL of mRNA (500 ng/µL) or siRNA (20 µM) was microinjected into the cytoplasm of zygotes. The injected zygotes were moved to KSOM medium and further cultured at 37 °C in a 5% CO_2_ atmosphere. The sequences of the siRNAs are listed in Supplementary Table S3.

### Histological Analysis of the Ovaries

Ovaries were collected and fixed in 4% PFA, embedded in paraffin, and cut into serial sections (3 µm). The sections were then stained with hematoxylin and eosin as reported previously.

### Immunofluorescence Staining and Image Acquisition

Ovaries were collected and fixed in 4% PFA overnight, gradient dehydrated through 10%, 20%, and 30% sucrose/PBS solution for 1 h each, embedded in optimal cutting temperature compound (Sakura, USA, 4583), frozen in liquid nitrogen, and cut into serial sections (8 µm) in a cryostat slicer. The sections were stored at −80 °C. Embryos were then fixed in 4% PFA in PBS at room temperature for 30 min.

Frozen sections and fixed embryos were permeabilized with 0.3% Triton X‐100 (Sigma, USA, T8787) at room temperature for another 30 min. The samples were then blocked with 1% BSA/PBS at room temperature for 1 h, incubated with primary antibodies at 4 °C overnight, and then incubated with Alexa Fluor 568‐conjugated goat anti‐rabbit (Thermo Fisher, USA, A11036), Alexa Fluor 488‐conjugated goat anti‐rabbit (Thermo Fisher, USA, A11034), Alexa Fluor 568‐conjugated donkey anti‐mouse (Thermo Fisher, USA, A10037), or Alexa Fluor 488‐conjugated goat anti‐mouse (Thermo Fisher, USA, A11001) secondary antibodies and DAPI (Thermo Fisher, USA, D1306) at room temperature for 1 h. The antibodies used in these experiments are listed in Supplementary Table S1. Between each step, the samples were rinsed three times with a washing solution (0.01% Triton X‐100 and 0.1% Tween‐20 in PBS). Image acquisition was performed with a Zeiss LSM800 confocal microscope system (Carl Zeiss, Germany).

### RNA Isolation and Gene Expression Analysis

Total RNA was isolated from 30 embryos in each group by using the RNeasy Plus Micro Kit (Qiagen, Germany, 74 034), and cDNA was synthesized from the total RNA by reverse transcription using the HiScript II 1st Strand cDNA Synthesis Kit (Vazyme, China, R211‐01) according to the manufacturer's instructions.

For *Acly* genotyping, single embryos were directly lysed in lysis buffer containing 2 µL 0.2% Triton X‐100 supplemented with 1 µL 10 µM oligo‐dT primer and 1 µL 10 mM dNTP mix after removing the zona pellucida with Tyrode's solution. Then, cDNA synthesis was performed by reverse transcription using the SuperScript II Reverse Transcriptase Kit (Invitrogen, USA, 18 064 014) according to the manufacturer's instructions. HiFi HotStart ReadyMix (KAPA, Switzerland, KK2602) was used to amplify the obtained cDNA.

Real‐time quantitative polymerase chain reaction (qPCR) was performed using SYBR Green Master Mix (Beyotime, China, D7268) in a Real‐Time PCR Detection System (Bio‐Rad, USA, CFX96). Primer sequences used to amplify the target genes are listed in Supplementary Table S4.

### RNA Sequencing (RNA‐seq) Library Preparation

A single 8‐cell stage embryo was collected as a sample. After removing the zona pellucida with Tyrode's solution (Sigma, USA, T1788), single‐embryo RNA samples were collected as described above. A total of 0.1 µL of External RNA Controls Consortium (ERCC) spike‐in RNA (Thermo Fisher, USA, 4456740) was added as an external control, and cDNA was acquired using the Smart‐seq2 method as previously described. Sequencing libraries were constructed by Annoroad Gene Technology Corporation (Beijing, China) using KAPA DNA HyperPlus (Roche, Switzerland, KK8514) according to the manufacturer's protocol. The samples were 125‐bp paired‐end sequenced on an Illumina HiSeq 2500 instrument.

### RNA‐seq Data Analysis

RNA‐seq data were processed using standard procedures, as previously reported.^[^ ^97^ ^]^ Briefly, the quality of the RNA‐seq data was checked using FastQC (v0.11.8). The raw reads were preprocessed using Trimmomatic (v0.35) to remove adapter sequences, and the clean reads were mapped to the mouse reference genome (mm10) using STAR (version 2.5.2a). The mapped reads were subsequently assembled into transcripts using Feature Counts (version 2.0.1). Gene counts were calculated using HTSeq (v0.6.1p1). The expression level of each gene was quantified using normalized fragments per kilobase of exon model per million mapped fragments (FPKM) and calibrated using ERCC. Differentially expressed genes (DEGs) were determined with the following threshold for significance: |log2 (fold change)| ≥ 1. The fold change was defined as si*Acly* (FPKM+1)/siNC (FPKM+1). Principal component analysis (PCA) and clustering analysis were performed using the FactoMineR R package. Gene Ontology (GO) term analysis, Kyoto Encyclopedia of Genes and Genomes (KEGG) analysis, and gene set enrichment analysis (GSEA) were performed using the ClusterProfiler R package. All visualizations, including Venn diagrams, scatter plots, and heatmaps, were performed using R software (version 4.3.3).

### CUT&Tag Library Preparation

The H3K27ac CUT&Tag assay was performed using the Hyperactive Universal CUT&Tag Assay Kit for Illumina (Vazyme, China, TD903). Embryos were incubated with 10 µL of prewashed ConA beads. Fifty microliters of antibody buffer and 2 µg of antibody were added, and the mixture was cultured overnight at 4 °C. Then, 50 µL digitonin (dig)‐wash buffer with secondary antibody was added, and the mixture was incubated at room temperature for 1 h. After washing three times with 200 µL dig‐wash buffer, 2 µL pG–Tn5 and 98 µL dig‐300 buffer were added; the samples were incubated at room temperature for 1 h and then washed twice with 200 µL dig‐wash buffer. Then we added 50 µL diluted TTBL buffer to the samples and incubated the samples at 37 °C for 1 h. The reaction was stopped with 5 µL proteinase K, 100 µL buffer L/B, and 20 µL DNA extract beads, which were all included with the kit. After extraction with phenol–chloroform and ethanol precipitation, PCR was performed to amplify the libraries under the following cycling conditions: 72 °C for 3 min, 95 °C for 3 min, 20 cycles of 98 °C for 10 s and 60 °C for 5 s, final extension at 72 °C for 1 min and hold at 4 °C. Post‐PCR clean‐up was performed by adding 100 µL DNA Clean Beads (Vazyme, China, N411). The libraries were incubated with the beads for 5 min at room temperature, washed gently in 80% ethanol, and eluted in 20 µL water. All libraries were sequenced using the Illumina NovaSeq PE150 platform according to the manufacturer's instructions.

### CUT&Tag Data Analysis

For the H3K27ac CUT&Tag read raw data, adapter trimming, genome mapping, and PCR duplicate removal were performed using Trim Galore, Bowtie2, and SAM tools, respectively. Paired‐end reads were aligned to the mm10 reference genome using Bowtie2 software with default parameters. The results were filtered using the SAMtools software with the following parameters: ‐bhS ‐q 20. BigWig files were generated using merged replicate BAM files with the bamCoverage tool in the deepTools software. The WashU Epigenome Browser (https://epigenomegateway.wustl.edu/browser/) was used to visualize H3K27ac CUT&Tag data. The macs2 callpeak function was used. Peak comparisons and overlaps were evaluated using BED Tools Suite for Autosomal Chromosomes. Genomic annotation of H3K27ac peaks was performed using the annotatePeak function of the ChIPseeker R package. H3K27ac peaks with a distance of < 2 kb were annotated using promoters. Distal H3K27ac peaks (distance to TSS > 3 kb) were assigned to enhancers. As histone H3K27ac is known to occur near promoters and enhancers, 2 kb windows for each promoter and enhancer were made to calculate the read coverage to measure the H3K27ac levels for different genes and then the results between the control and treatment groups were compared. Aberrant H3K27ac peaks were identified as those with fold change of read density (si*Acly*/siNC) ≥ 1.5.

### Statistical Analysis

All statistical analyses were performed using GraphPad Prism (version 8.0). Details of individual tests, including the number and type of replication performed (n) and the reported error as mean ± S.D., are outlined within each Figure legend. Statistical significance is indicated as *P < 0.05, **P < 0.001, or ***P < 0.001, as calculated by a two‐tailed unpaired Student's *t*‐test for comparisons between two groups, and one‐way ANOVA with Dunn's multiple comparison test or Sidak's multiple comparison test for comparisons of more than two groups.

### Ethical Statement

This study was approved by the Ethics Committee of Sir Run Run Shaw Hospital, Zhejiang University School of Medicine (Project No. Y21H040019, Approval No. 20210319‐32), and the Institutional Animal Care and Use Committee (IACUC) of Zhejiang University.

## Conflict of Interest

The authors declare no conflict of interest.

## Author Contributions

Y.M., Y.Z., W.Y., and Y.L.Z. contributed equally to this work. Songying Zhang, Yin‐Li Zhang, and Heng‐Yu Fan conceived the project and designed research. Yerong Ma, Yingyi Zhang, Weijie Yang, Siya Liu, Zhanhong Hu, Yan Zhou, Peipei Ren, Mengjia Qiu, and Jiamin Jin performed the experiments and analyzed the data. Yerong Ma, Yingyi Zhang, Weijie Yang, Huifang Jiang, Fei Huang, and Liujian Ouyang performed sequencing data analyses. Xiaomei Tongand Feng Zhou contributed to the collection of human embryos. Yerong Ma, Yingyi Zhang, Weijie Yang and Yin‐Li Zhang wrote the manuscript.

## Supporting information



Supporting Information

## Data Availability

All RNA‐seq and CUT&Tag datasets generated in this work have been deposited into the Sequence Read Archive (SRA) database (PRJNA1045095, PRJNA1149439). The data that support the findings of this study are available in the supplementary material of this article.
