# Supplementary material for: The Nuclear Localization of ACLY Guards Early Embryo Development Through Recruiting P300 and HAT1 to Promote Histone Acetylation and Transcription
Source: Adv Sci (Weinh). 2025 Jun 5;12(31):e14367. doi: 10.1002/advs.202414367 (PMC12376542; doi:10.1002/advs.202414367)
Supplement: Supplementary file 1 — Supporting Information [file ADVS-12-e14367-s001.docx]

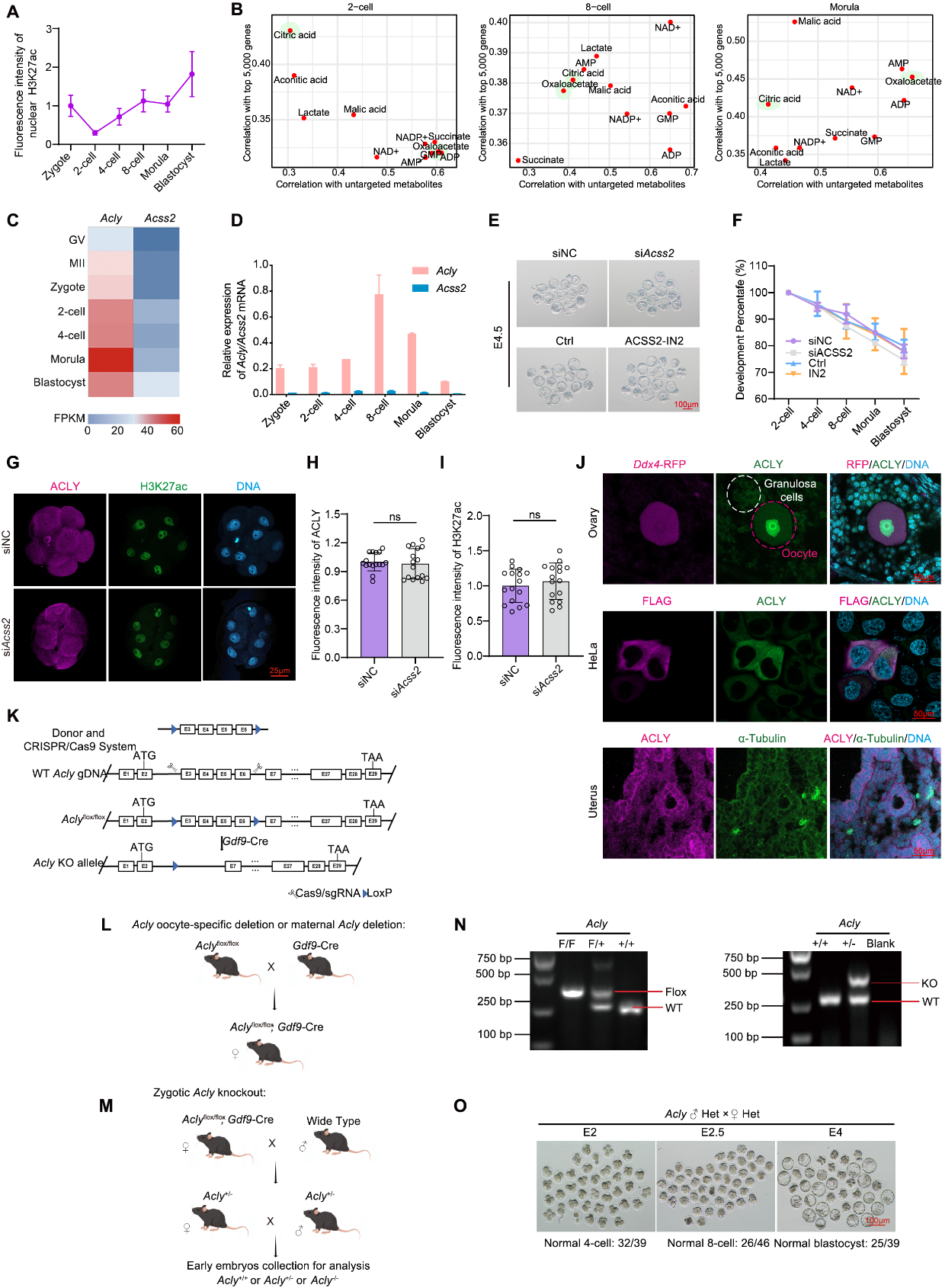


**Figure S1 ACLY is highly expressed in the nuclei of mouse oocytes and embryos.**

1. Quantification of H3K27ac fluorescence intensity (normalized to DAPI) in embryos from zygote to blastocyst stages (n=16 embryos per stage). **(B)** The correlation analysis of top 5000 transcription active genes with untargeted metabolites at 2-cell, 8-cell, or morula stage in mouse. **(C)** Heatmap of *ACLY* and *ACSS2* expression in human embryos at different stages, based on previously published RNA-seq data. **(D)** RT‒PCR of *Acly* (pink) and *Acss2* (blue) expression in embryos at different stages (n=2 biological replicates; 30 embryos per replicate), normalized to *Actin* and *Gapdh*. **(E)** Representative images of embryos at E4.5 after *Acss2* KD (si*Acss2*) or pharmacological inhibition of ACSS2 with 5 μM ACSS2-IN2. Scale bar = 100 μm. **(F)** The development percentage at each stage for the embryos after *Acss2* KD (si*Acss2*) or pharmacological inhibition (ACSS2-IN2) (5 μM) inhibition (n=48, siNC; n=48, si*Acss2*; n=60, Ctrl; n=64, ACSS2-IN2). **(G-I)** Representative images (G) and qualitative analysis (H-I) of 8-cell stage embryos stained with anti-ACLY (violet) antibody and anti-H3K27ac (green) antibody in control and *Acss2* knockdown (KD) embryos (n=16). Nuclei were stained with DAPI (light blue). Scale bar = 25 μm. **(J)** Representative images of follicles from *Ddx4*-RFP mice, or HeLa cells transfected with Flag-ACLY plasmid, and uterus tissue stained with anti-ACLY and anti-FLAG, or anti-α-Tubulin antibody (n=3). Nuclei were stained with DAPI (light blue). Scale bar = 50 μm. **(K)** Genetic strategy for *Acly* conditional (floxed allele) and systemic knockout (KO) mice. **(L-M)** Crossing strategy for obtaining *Acly* maternal knockout mice (L) and systemic knockout mice (M). **(N)** Genotype identification of *Acly^flox/flox^*, *Acly^flox/+^*, *Acly ^+/+^*, or *Acly ^+/-^* mice. **(O)** Representative images of E2, E2.5, and E4 embryos obtained from mating heterozygous (*Acly*^+/-^) mice (n=3). Student’s t test (F, H, and I).

**
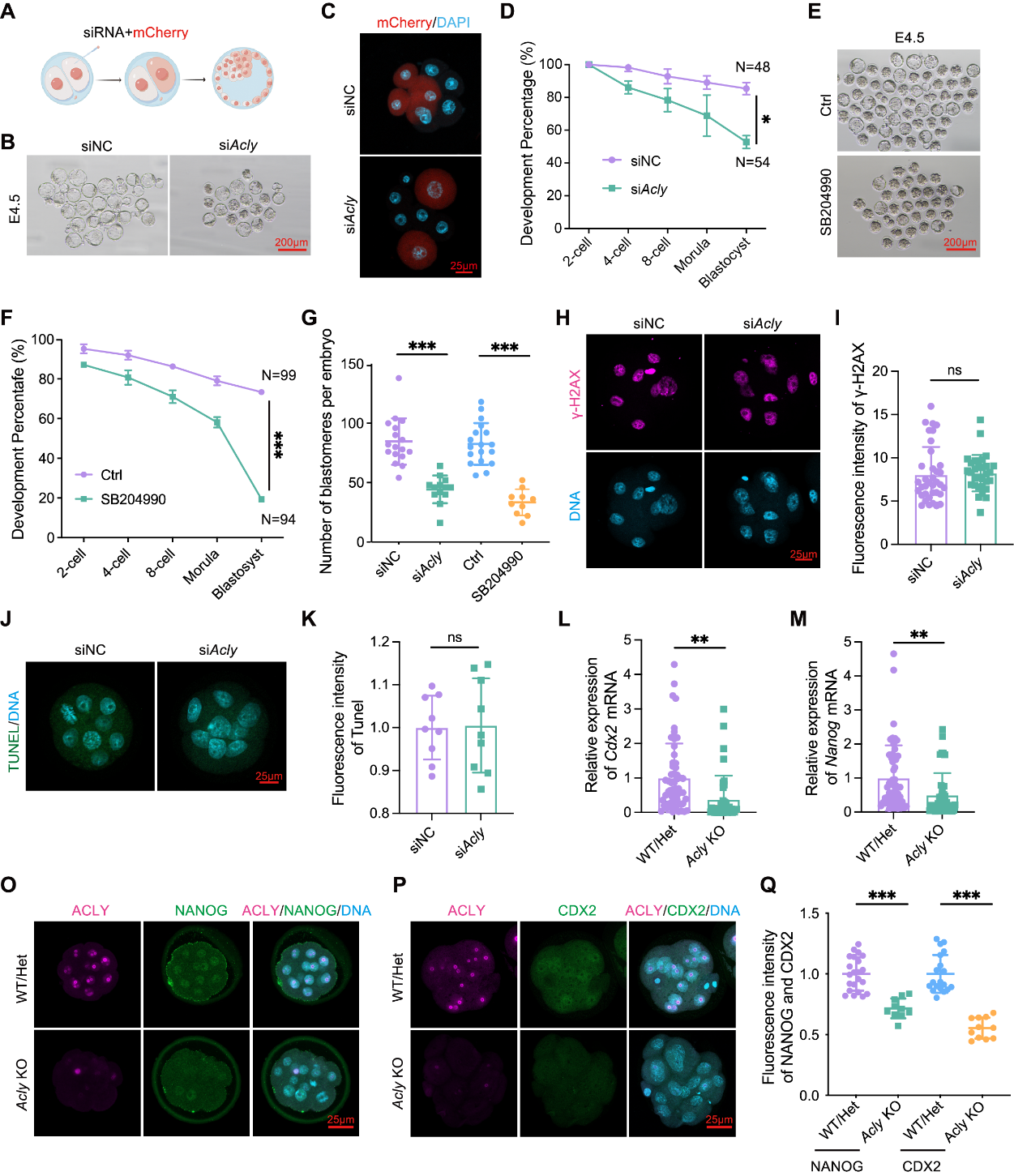
**

**Figure S2. Zygotic *Acly* deletion impairs blastocyst formation and cell proliferation without triggering DNA damage or apoptosis.**

1. Schematic of blastomere-specific siRNA microinjection at the 2-cell stage. **(B)** The development process of embryos that developed from 2-cell stage embryos injected with 20 μM siNC or si*Acly* and 200ng/μL *mCherry* mRNA (n=3). Scale bar = 200 μm. **(C)** Representative images of E2.5 embryos after injection with siNC+mCherry or si*Acly+*mCherry in one of the blastomeres at the 2-cell stage (n=10). Nuclei were stained with DAPI (light blue). Scale bar = 25 μm. **(D)** The development percentage at each stage of embryos post-2-cell injection (n=48, siNC; n=54, si*Acly*). **(E-F)** The development process (E) and development percentage (F) at each stage of embryos treated with 20 μM DMSO or SB204990 at the zygote stage (n=99, Ctrl; n=94, SB204990). Scale bar = 200 μm. **(G)** Reduced blastomere numbers in *Acly*-perturbed blastocysts (n=17, siNC; n=13, si*Acly*; n=18, Ctrl; n=10, SB204990). **(H-K)** Representative images (H and J) and statistical analysis (I and K) of the fluorescence intensity of E2.5 embryos after injection with siNC or si*Acly* stained with γ-H2AX (violet) or TUNEL (green) (n=36, siNC, n=31, si*Acly*, γ-H2AX; n=9, siNC, n=9, si*Acly*, TUNEL). Nuclei were stained with DAPI (light blue). Scale bar = 25 μm. **(L-M)** RT‒qPCR results of *Cdx2* (L) and *Nanog* (M) expression in 8-cell stage embryos, normalized to *Actin* and *Gapdh*. The WT/Het and KO groups were divided according to the expression of *Acly* (n=54, WT/Het; n=40, KO). **(O-P)** Representative images of WT and KO embryos stained with anti-ACLY (violet), anti-NANOG (O), or anti-CDX2 (P) (green) antibodies (n=36, WT/Het; n=10, KO) co-stained with DAPI (light blue). Scale bar = 25 μm. **(Q)** Quantification of the fluorescence intensity of NANOG and CDX2 (n=22/19, WT/Het; n=19/11, KO). Data are presented as mean ± S.D. *NS* *p*>0.05; ** *p*<0.01; *** *p*<0.001. Student’s t test (D, F, G, I, K, L, M and Q).


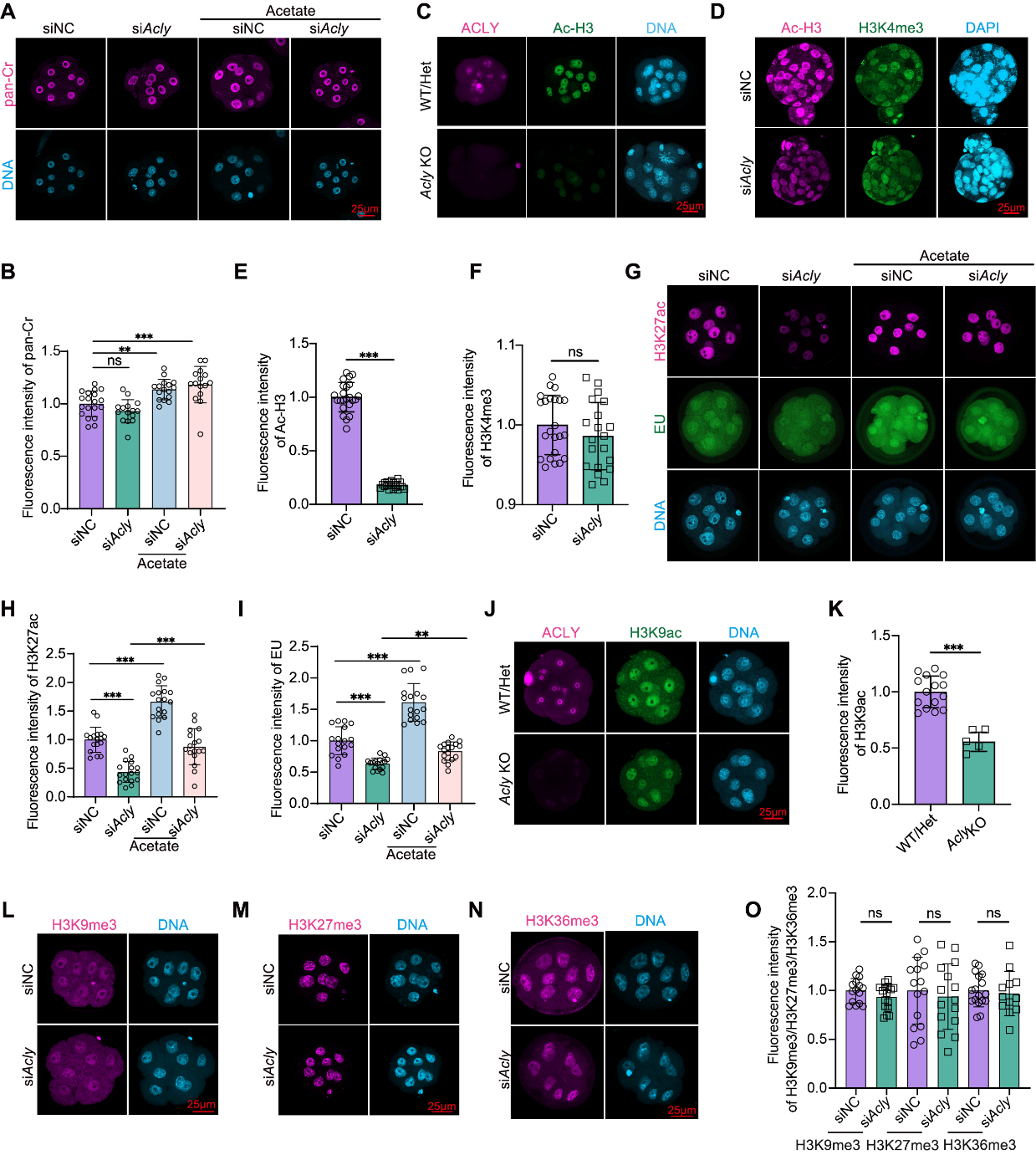


**Figure S3. Zygotic ACLY loss selectively reduces histone acetylation without altering repressive methylation marks.**

1. **B)** Representative images (A) and quantitative analysis (B) of 8-cell embryos stained with anti-ACSS2 (green) after *Acly*-knocking down and cultured with or without 2mM acetate (n=18). Scale bars = 25 μm. Data are presented as mean ± S.D. **(C)** Representative images of 8-cell stage embryos stained with anti-ACLY (violet) antibody and anti-Ac-H3 (green) antibody (n=16, WT/Het; n=5, KO). Embryos were obtained from mating *Acly*^+/-^ female and male mice. Nuclei were stained with DAPI (light blue). Scale bar = 25 μm. **(D)** Representative images of blastocysts developed from injected zygotes, stained with anti-Ac-H3 (violet) antibody and anti-H3K4me3 (green) antibody (n=25) post-*Acly* knockdown (KD). Nuclei were stained with DAPI (light blue). Scale bar = 25 μm. **(E-F)** Statistical analysis of the fluorescence intensity of ac-H3 (E) and H3K4me3 (F) post-*Acly* KD (n=23, si*NC*; n=20, si*Acly*). **(G-I)** Representative images (G) and qualitative analysis (H-I) of 8-cell embryos stained with anti-H3K27ac (violet) antibody and EU staining after treatment (n=17). Nuclei were stained with DAPI (light blue). Scale bar = 25 μm. **(J-K)** Representative images (J) and quantitative analysis (K) of 8-cell stage embryos stained with anti-ACLY (violet) antibody and anti-H3K9ac (green) antibody (n=15, WT/Het; n=7, KO). Embryos were obtained from mating *Acly*^+/-^ female and male mice. Nuclei were stained with DAPI (light blue). Scale bar = 25 μm. **(L-O)** Representative images (L-N) and quantitative analysis (O) of 8-cell stage embryos stained with anti-H3K9me3 (L), or H3K27me3 (M), or H3K36me3 (N) (violet) antibody post-*Acly* KD (n=15/15/17, siNC; n=16/15/12, si*Acly*). Nuclei were stained with DAPI (light blue). Scale bar = 25 μm. Data are presented as mean ± S.D. *NS* *p*>0.05; *** *p*<0.001. Student’s t test (E, F, K, and O). One-way ANOVA (B, H, and I).
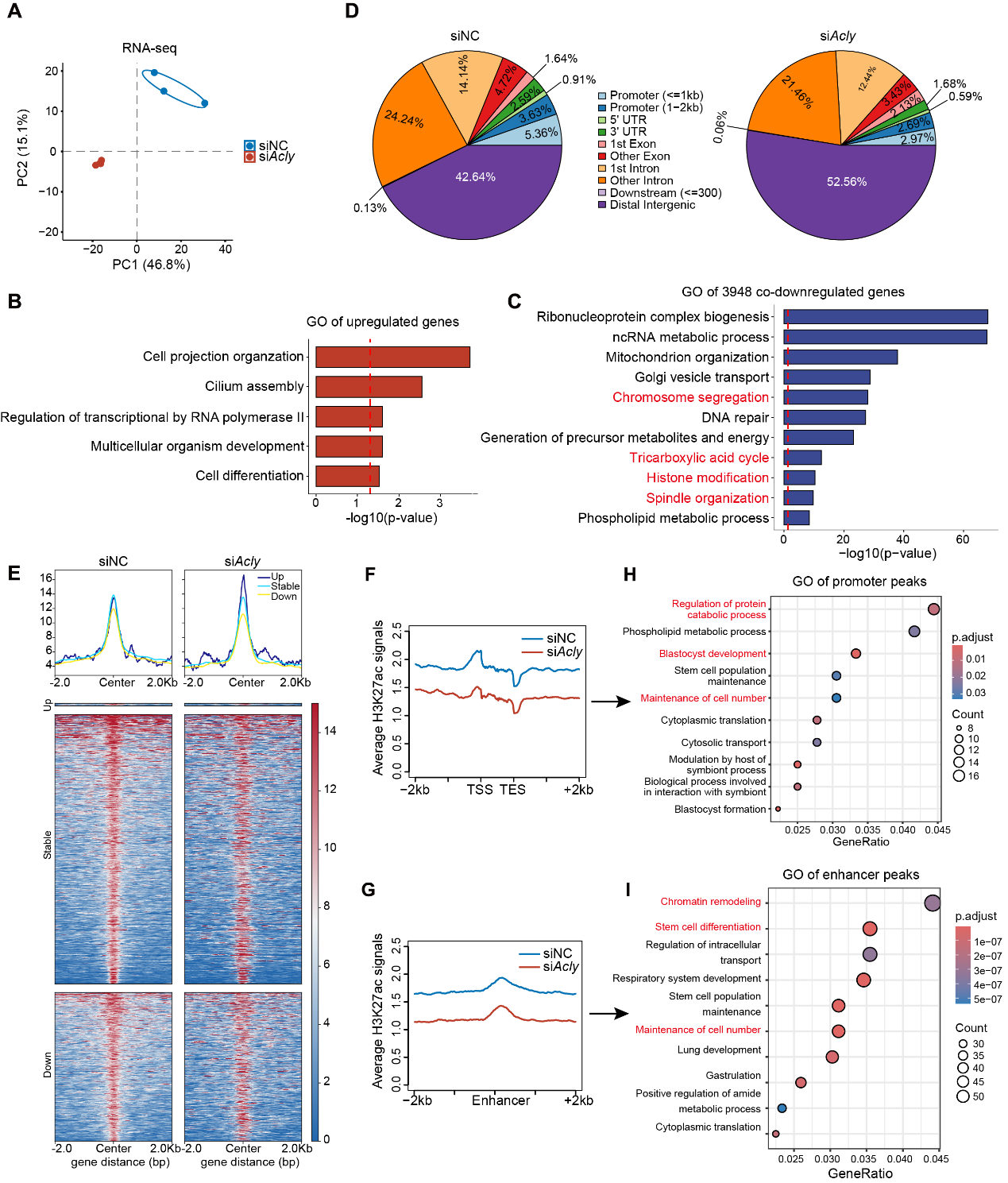


**Figure S4. Zygotic *Acly* deletion impairs H3K27ac establishment and transcription.**

1. Principal component analysis (PCA) of RNA-seq profiles from *Acly* knockdown (KD, si*Acly*) vs. control (siNC) 8-cell stage embryos. **(B)** Gene Ontology (GO) enrichment for 240 genes that were upregulated in *Acly* KO or *Acly* KD 8-cell stage embryos. **(C)** GO enrichment for 3948 both downregulated genes in *Acly* KO and *Acly* KD 8-cell stage embryos. **(D)** Genomic distribution of H3K27ac CUT&Tag peaks in siNC and si*Acly* 8-cell stage embryos (n=2). **(E)** H3K27ac signal metaplots at genes stratified by RNA-seq expression changes (Up/Down/Stable). Peaks assigned to nearest gene. **(F-G)** The meta plots showed the downregulation of H3K27ac signals post *Acly* KD around the promoter (F) or enhancer (G) (± 2 kb) (n=2). **(H-I)** GO terms enriched for genes linked to downregulated promoter- (H) or enhancer- (I) associated H3K27ac peaks.
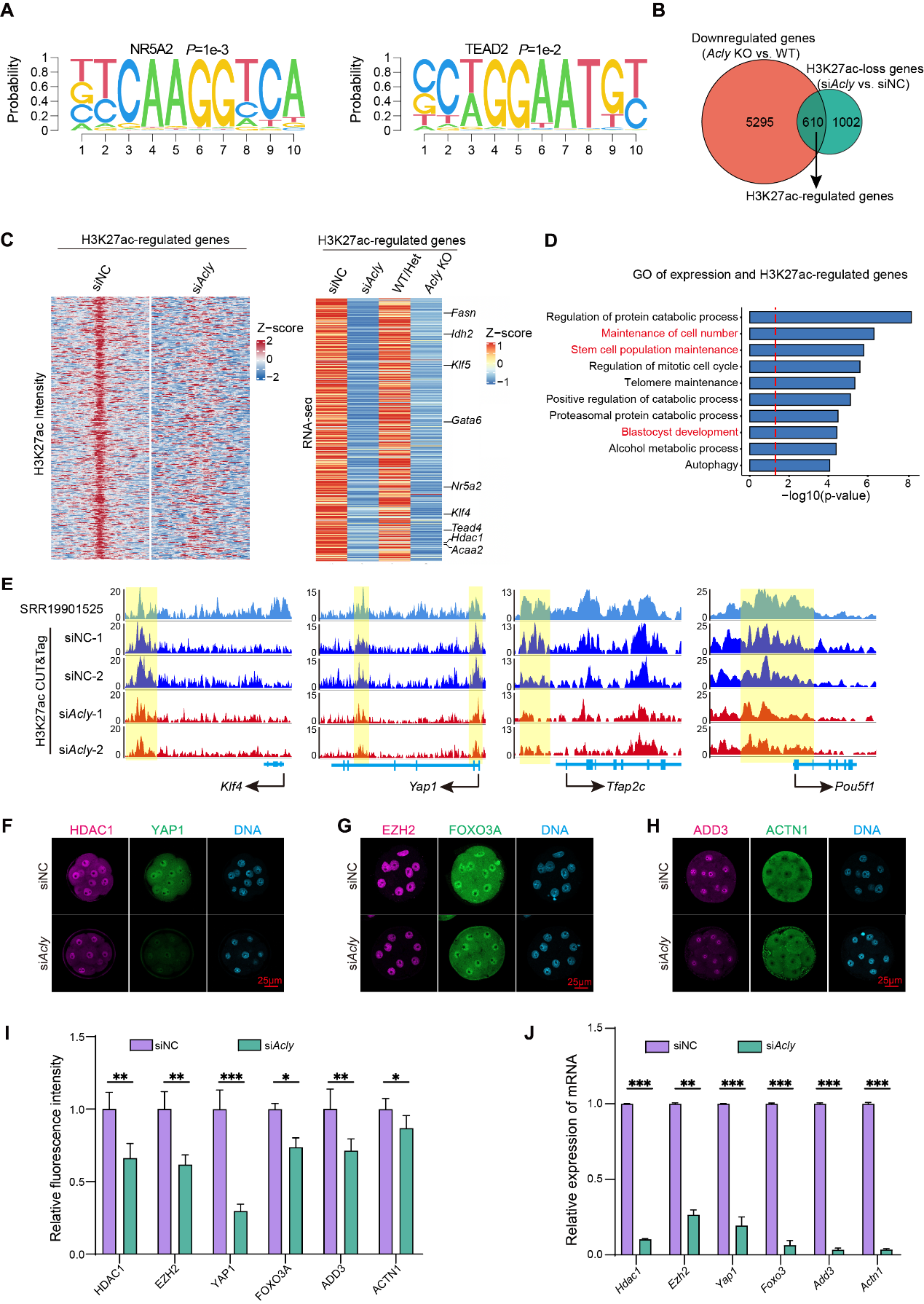


**Figure S5. Zygotic *Acly* deletion impairs H3K27ac establishment and transcription.**

1. De novo motif enrichment for transcription factors (TFs) associated with upregulated H3K27ac peaks. **(B)** The Venn diagram shows overlapping downregulated genes in the *Acly* KO RNA-seq and H3K27ac-loss genes post *Acly* knocking down (KD). **(C)** Integrated heatmap of H3K27ac signals (left) and RNA-seq expression (right) for 610 co-downregulated genes in *Acly* KO/KD embryos. **(D)** The biological process GO terms enriched of 610 H3K27ac-dependent co-downregulated genes **(E)** Genome browser view showing the H3K27ac signals at the *Klf4*, *Yap1*, *Tfap2c*, and *Pou5f1* genes in wild-type (wathet blue, SRR19901525), siNC (blue) and si*Acly* (red) 8-cell stage embryos. **(F-I)** Representative images (F-H) and quantitative analysis (I) of 8-cell stage embryos stained with anti-HDAC1 (F) / EZH2 (G) / ADD3 (H) (violet) and anti-YAP1 (F) /FOXO3A (G) /ACTN1 (H) (green) antibodies post *Acly* KD (n=15, siNC; n=15, si*Acly*). Nuclei were stained with DAPI (light blue). Scale bar = 25 μm. **(J)** RT‒qPCR results of *Hdac1*/*Ezh2*/*Foxo3*/*Yap1*/*Add3*/*Actn1* expression in 30 8-cell stage embryos post *Acly* KD, normalized to *Actin* and *Gapdh*. Data are presented as mean ± S.D. * *p*<0.05; ** *p*<0.01, *** *p*<0.001. Student’s t test (I, and J).


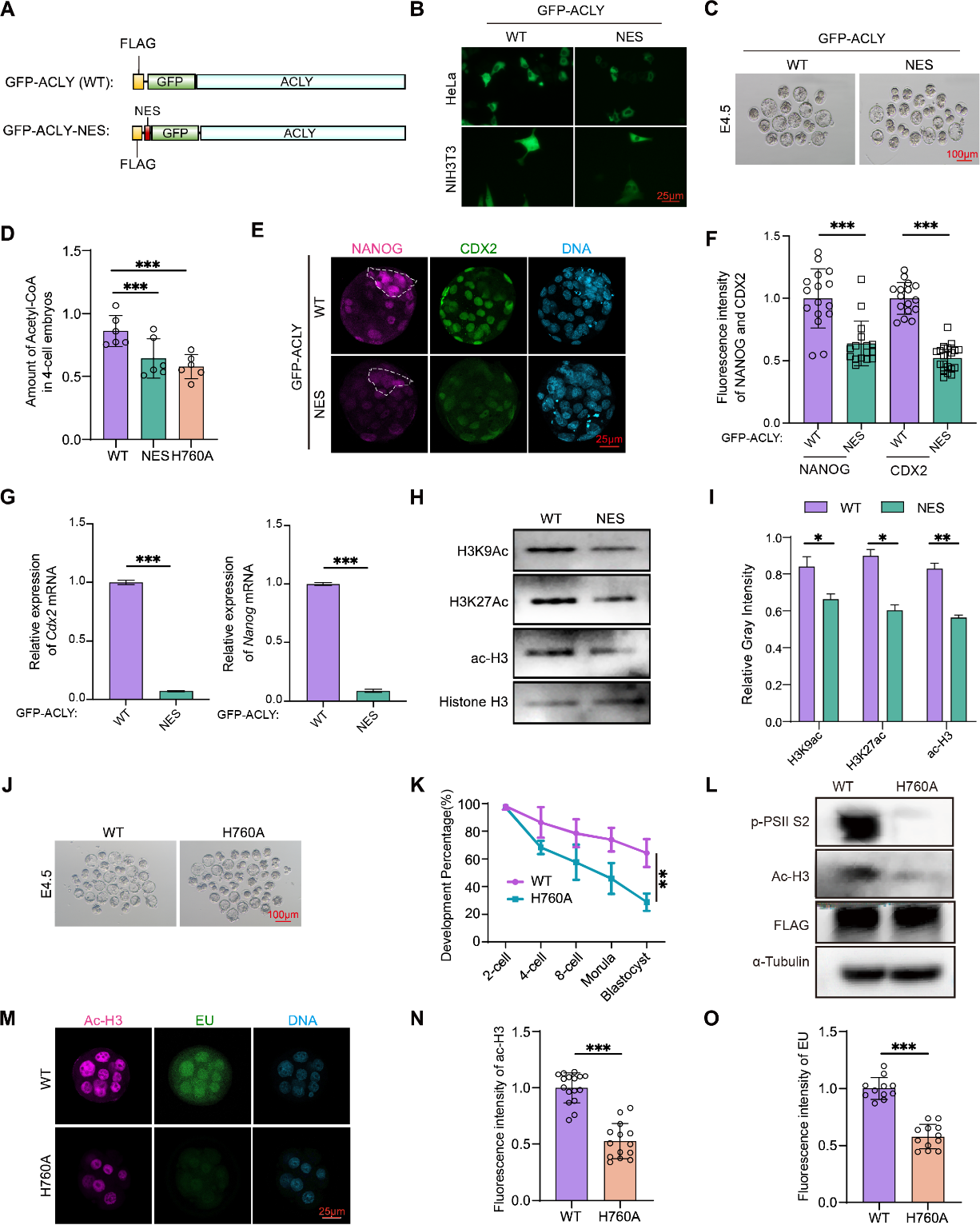


**Figure S6. Nuclear localization and catalytic activity of ACLY is required for early embryo development.**

1. Diagram of the plasmid constructed for exporting ACLY out of the nucleus. NES, nuclear export sequence. **(B)** Subcellular localization of GFP-tagged ACLY-WT and ACLY-NES in HeLa/NIH3T3 cells. Scale bar = 25 μm. **(C)** The development process of embryos developed from zygotes injected with GFP, GFP-*Acly*-WT mRNAs, or GFP-*Acly*-NES mRNAs. **(D)** Acetyl-CoA content in 500 4-cell post-injection (n=6). **(E-F)** Representative images (E) and quantitative analysis (F) of the fluorescence intensity of blastocysts stained with anti-NANOG (violet) and anti-CDX2 (green) antibodies, developed from injected zygotes (n=16, GFP-*Acly*-WT; n=20, GFP-*Acly*-NES). The highlighted areas were inner cell mass. Nuclei were stained with DAPI (light blue). Scale bar = 25 μm. **(G)** RT‒qPCR results of *Cdx2* (left) and *Nanog* (right) expression of blastocysts developed from injected zygotes, normalized to *Actin* and *Gapdh*. **(H-I)** Western blot (H) and statistical result (I) of 150 E4.5 embryos after different treatment (n=2). α-Tubulin served as a loading control. **(J-K)** The development process (J) and development percentage at each stage at each stage (K) for the embryos that developed from zygotes injected with 500 ng/μL *Acly*^WT^ or *Acly*^H760A^ mRNA. Scale bar = 100 μm. Data are presented as mean ± S.D.  **(L)** Western blot of 150 E4.5 embryos after different treatment. α-Tubulin served as a loading control. **(M-O)** Representative images (M) and quantitative analysis (N-O) of 8-cell developed from injected zygotes, stained with anti-ac-H3 (violet) antibody and EU staining (n=24). Nuclei were stained with DAPI (light blue). Scale bar = 25 μm. Data are presented as mean ± S.D. * *p*<0.05; ** *p*<0.01, *** *p*<0.001. Student’s t test (D, F, G, I, N, and O).
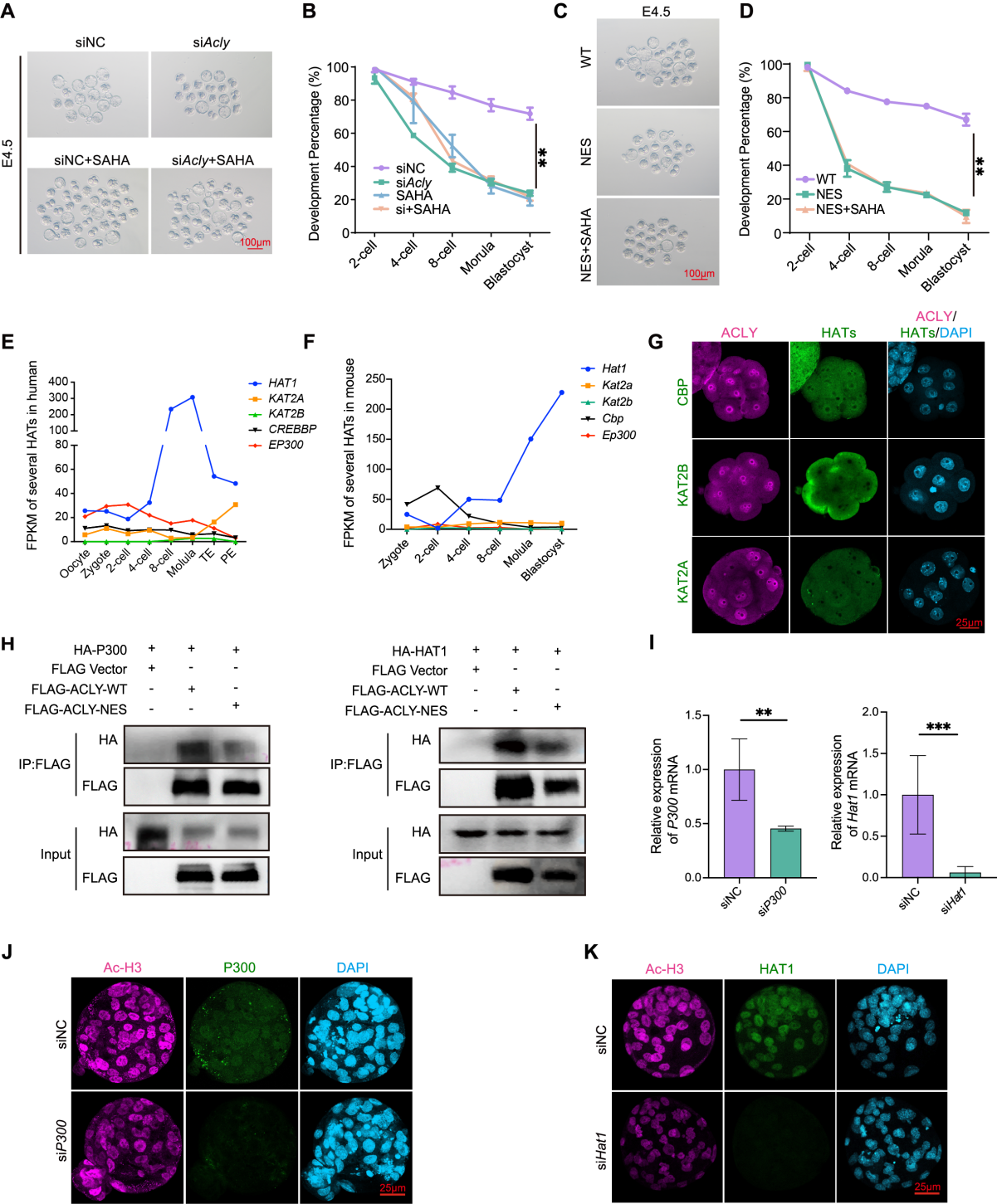


**Figure S7. Functional interplay between ACLY and HATs (P300/HAT1) coordinates histone acetylation to regulate early embryo development.**

**(A-B)** The development process (A) and development percentage at each stage (B) at each stage for the embryos that developed from zygotes injected with 20μM siNC or si*Acly*, and cultured with or without 0.05μM SAHA. Scale bar = 100μm. Data are presented as mean ± S.D. ** *p*<0.01 (One-way ANOVA). **(C-D)** The development process (C) and development percentage at each stage (D) at each stage for the embryos that developed from zygotes injected with 500 ng/μL *Acly*^WT^ or *Acly*^NES^ mRNA, and cultured with or without 0.05μM SAHA. Scale bar = 100μm. Data are presented as mean ± S.D. **(E-F)** The expression levels of several HATs obtained from the RNA-seq results of human (E) and mouse (F) embryos at different stages. **(G)** Representative images of WT embryos stained with anti-ACLY (violet) antibody and anti-CBP, or anti-KAT2A, or anti-KAT2B antibodies. Nuclei were stained with DAPI (light blue). Scale bar = 25 μm. **(H)** Exogenous ACLY binds with P300 and HAT1 according to the coimmunoprecipitation (Co-IP) results using HEK293T. **(I)** RT‒qPCR results of *P300* (*p*=0.0286) and *Hat1* (*p*=0.0022) expression in 30 morulae after *P300* or *Hat1* knockdown (KD)*.* **(J-K)** Representative images of blastocysts stained with anti-Ac-H3 (violet) antibody and anti-P300 (J) and anti-HAT1 (K) (green) antibodies after *P300* or *Hat1* KD*.* (n=15). Nuclei were stained with DAPI (light blue). Scale bar = 25 μm. ** *p*<0.01, *** *p*<0.001. Student’s t test (I). One-way ANOVA (B, and D).


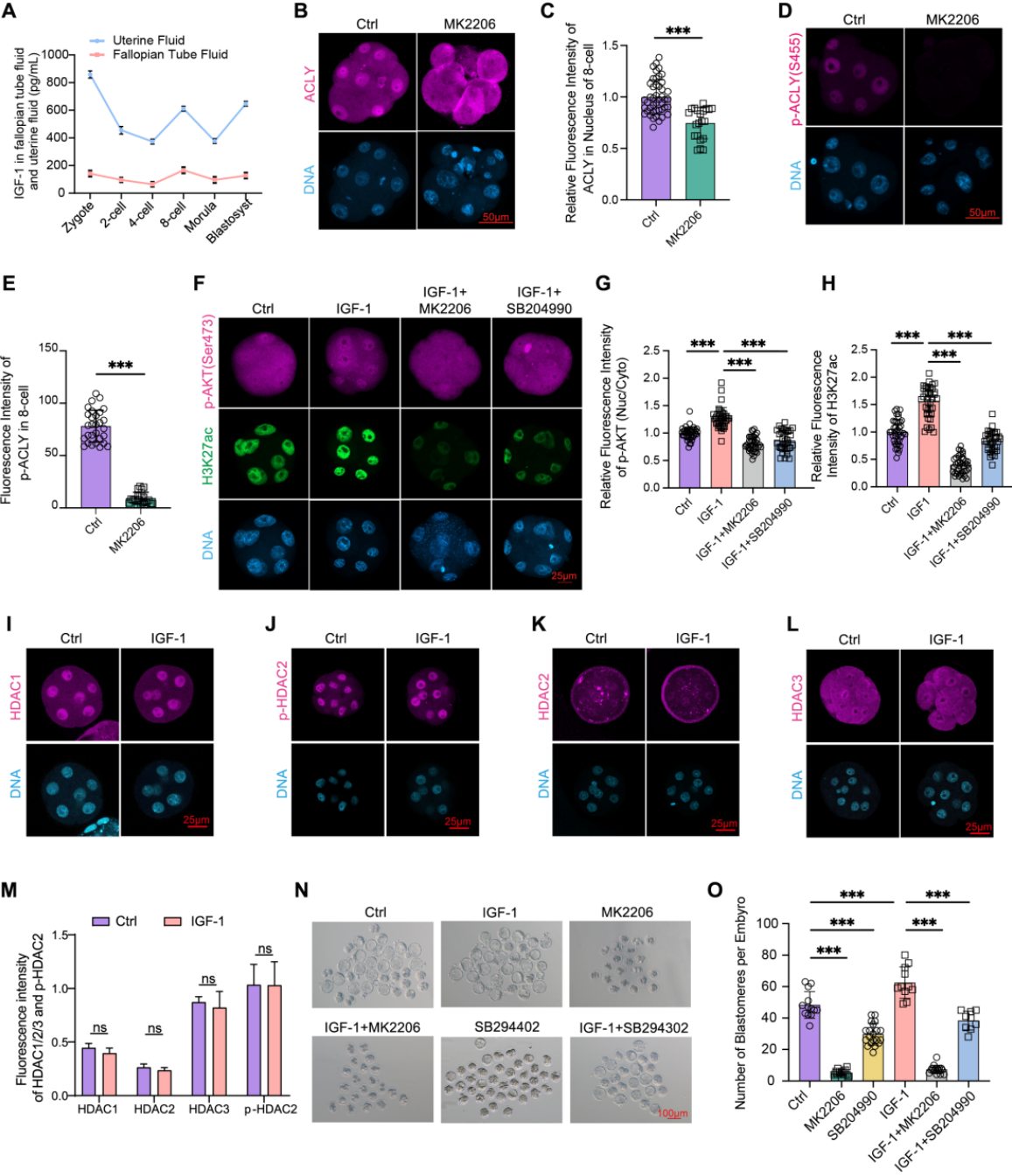


**Figure S8. AKT pathway facilitates ACLY phosphorylation and the nuclear localization of ACLY and P300/HAT1 without affecting HDACs.**

**(A)** IGF-1 concentrations in uterine and fallopian tube fluids dynamically fluctuate across early embryo developmental stages (zygote to blastocyst), as measured by ELISA in triplicate samples pooled from three mice per time point. Zygote: 20–22 h post-hCG; 2-cell: 46–48 h post-hCG; 4-cell: 54–56 h post-hCG; 8-cell: 68–70 h post-hCG; Morula: 76-78 h post-hCG; Blastocyst: 96 h post-hCG. **(B-C)** Representative images (B) and quantitative analysis (C) of the fluorescence intensity in nucleus of 8-cell stage embryos stained with anti-ACLY (violet) antibody treated with 10 μM DMSO or MK2206 (n=45, Ctrl; n=21, MK2206). Nuclei were stained with DAPI (light blue). Scale bar = 50 μm. **(D-E)** Representative images (D) and quantitative analysis (E) of the fluorescence intensity of 8-cell stage embryos stained with anti-p-ACLY (Ser455) (violet) antibody treated with 10 μM DMSO or MK2206 (n=29, Ctrl; n=21, MK2206). Nuclei were stained with DAPI (light blue). Scale bar = 50 μm. **(F-H)** Representative images (F) and quantitative analysis (G-H) of embryos stained with anti-p-AKT (Ser473) (violet) and H3K27ac (green) antibodies treated with 10 μM DMSO, 10ug/ml IGF-1, both IGF-1 and MK2206, or both IGF-1 and 20 μM SB204990. Nuclei were stained with DAPI (light blue). Scale bar = 25 μm (n=41, Ctrl; n=38, IGF-1; n=39, both IGF-1 and MK2206; n=36, both IGF-1 and SB204990). **(I-L)** Representative immunofluorescence images of 8-cell stage embryos stained with ainti-HDAC1 (I), p-HDAC2 (Ser394) (J), HDAC2 (K), or HDAC3 (L) antibody treated with or without 10ug/ml IGF-1 (n=15 embryos per group). Nuclei were stained with DAPI (light blue). Scale bar = 25 μm. **(M)** Quantitative analysis of the fluorescence intensity of HDAC1, HDAC2, HDAC3, and p-HDAC2 (Ser394) at 8-cell stage embryos treated with or without 10ug/ml IGF-1 (n=15 embryos per group). Data are presented as mean ± S.D. **(N-O)** The embryo development (N) and quantitative analysis of the number of blastomeres in each blastocyst (O) after indicated drugs treatment (n=13, Ctrl; n= 16, MK2206; n=20, SB204990; n=11, IGF-1; n=16, both IGF-1 and MK2206; n=9, both IGF-1 and SB204990). Scale bar =100 μm. Data are presented as mean ± S.D. *NS* *p*>0.05; * *p*<0.05; ** *p*<0.01, *** *p*<0.001. Student’s t test (C, and E). One-way ANOVA (G, H, M, and O).


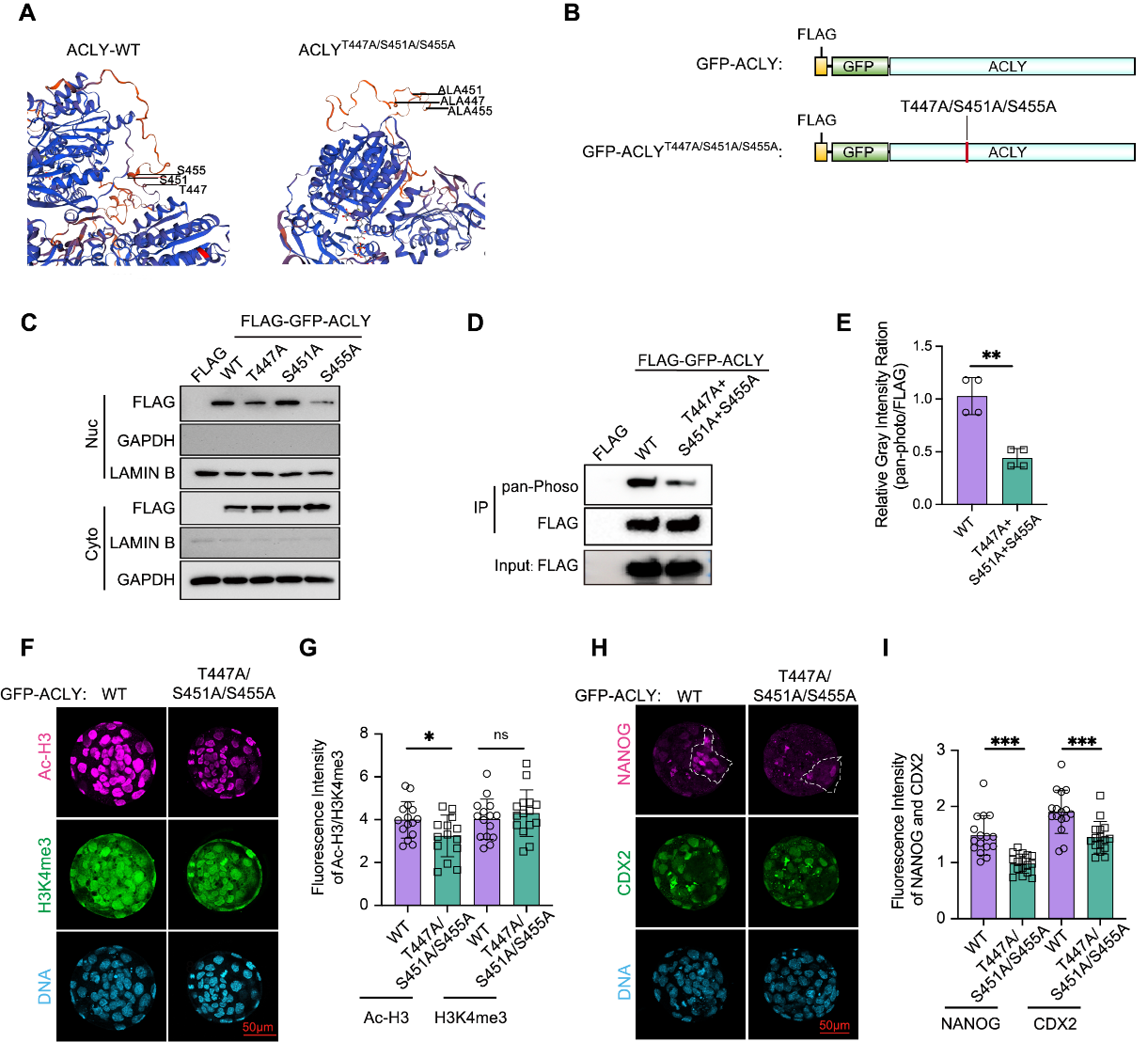


**Figure S9. Phosphorylation of Thr447, Ser451, and Ser455 in ACLY is responsible for its nuclear localization.**

1. Predicted structures of wild-type (WT) ACLY and its phospho-deficient mutant (T447A/S451A/S455A) generated using SWISS-MODEL (expasy.org). **(B)** Schematic of the plasmid construct for sustained inactivation of T447, S451, and S455 sites. (**C)** Western blot of nucleocytoplasmic distribution of ACLY mutants in transfected HeLa cells. **(D-E)** The western blot (D) and statistical result (E) of the phosphorylation level of ACLY in HeLa cells expressing WT or mutant plasmids (*p*=0.0010). **(F-G)** Representative images (F) and quantitative analysis (G) of blastocysts stained with anti-Ac-H3 (violet) and anti-H3K4me3 (green) antibodies, developed from injected zygotes (n=15, GFP-ACLY (WT); n=15, GFP-ACLY^T447A/S45A/S455A^; Ac-H3: *p*=0.0325; H3K4me3: *p*=0.4511). Nuclei were stained with DAPI (light blue). Scale bar = 25 μm. **(H-I)** Representative images (H) and quantitative analysis (I) of blastocysts stained with anti-NANOG (violet) and anti-CDX2 (green) antibodies, developed from injected zygotes (n=16, GFP-ACLY (WT); n=16, GFP-ACLY^T44A7/S45A/S455A^). The highlighted areas were inner cell mass. Nuclei were stained with DAPI (light blue). Scale bar = 25 μm. Data are presented as mean ± S.D. *ns,* *p*>0.05; * *p*<0.05; ** *p*<0.01; *** *p*<0.001. Student’s t test (E, G, and I).


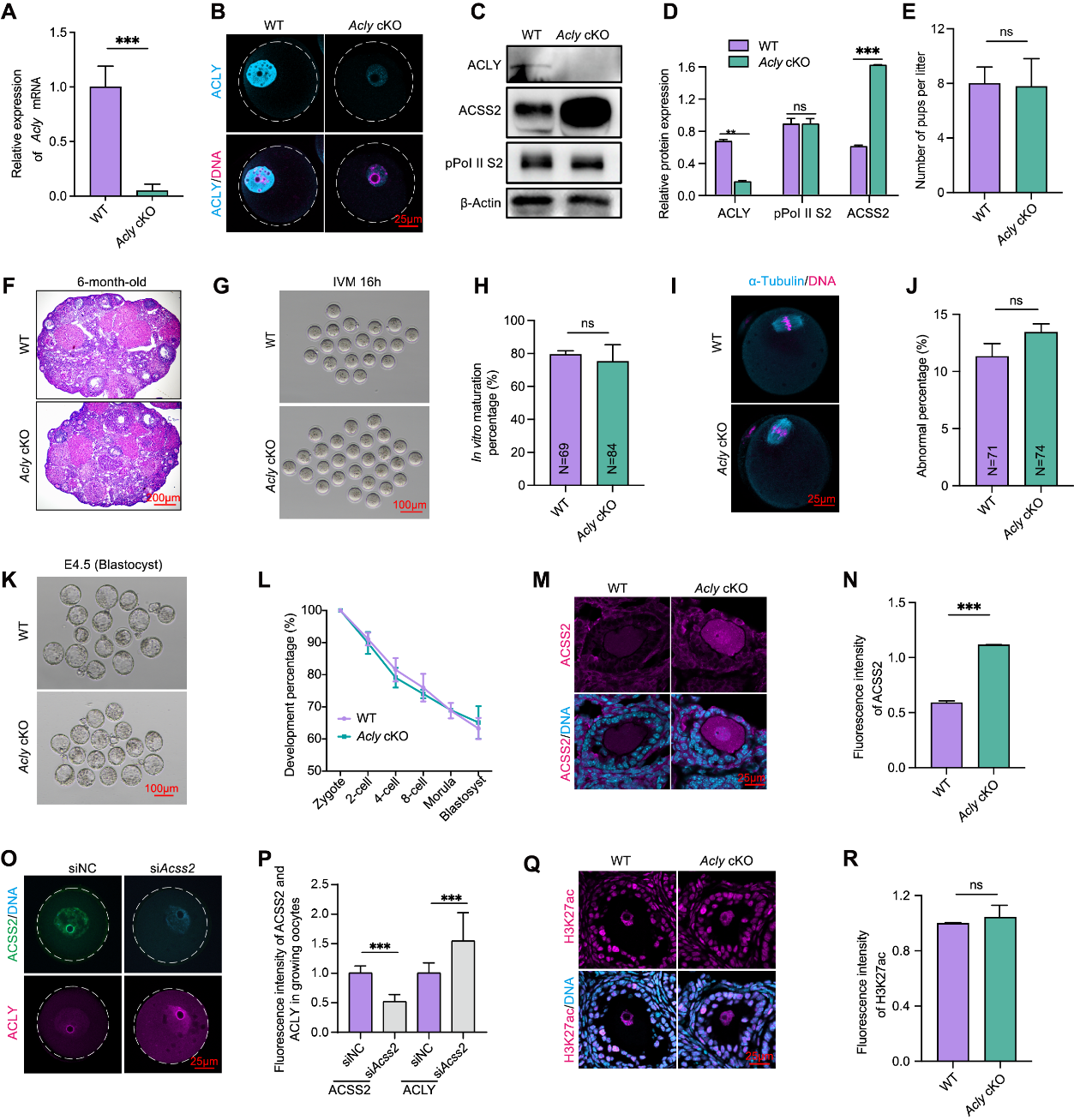


**Figure S10. Maternal ACLY deletion in oocytes did not affect follicle development and oocyte maturation.**

**(A)** *Acly* mRNA levels in GV oocytes from WT and *Acly* cKO mice (RT‒qPCR normalized to *Actin* and *Gapdh*; n = 6, WT; n=5, *Acly* cKO) (n=6, WT; n=5, *Acly* cKO). **(B)** Representative images of ACLY protein expression in GV-stage oocytes obtained from WT and *Acly* cKO mice. Oocytes were stained with anti-ACLY (light blue) antibody (n=10) co-stained with DAPI (violet). Scale bar = 25 μm. **(C)** Western blot of 100 growing oocytes from WT or *Acly* cKO female mice (n=2). β-Actin served as a loading control. **(D)** Statistical analyses of western-blot using 100 growing oocytes from WT or *Acly* cKO female mice (n=2). **(E)** Statistical analysis of the average litter size of 6 WT and *Acly* cKO female mice mated with wild-type male mice over 6 months (n=3). **(F)** HE staining images of ovaries from 6-month-old mice (n=2). Scale bar = 200 μm. **(G-H)** Representative images (G) and statistical analyses (H) of the mature oocytes obtained *in vivo* after superovulation (n=69, WT; n=84, *Acly* cKO). Scale bar =100 μm. **(I-J)** Representative images (I) and percentage of abnormal spindle morphology (J) of mature oocytes at the MII stage, obtained after superovulation *in vivo* and stained with anti-α-Tubulin (light blue) antibody and DAPI (violet) for chromosome indication (n=71, WT; n=74, *Acly* cKO). **(K-L)** The *in vitro* embryo development process (K) and development percentage (L) of zygotes obtained from WT or *Acly* cKO female mice mated with WT male mice (n=55, WT; n=58, *Acly* cKO). Scale bar = 100 μm. **(M)** Representative images of growing follicles stained with anti-ACSS2 (violet) antibody from WT and *Acly* cKO mice (n=3). Nuclei were stained with DAPI (light blue). Scale bar =25 μm. **(N)** Qualitative analysis of the fluorescence intensity of growing follicles stained with anti-ACSS2 antibody from WT and *Acly* cKO mice. **(O-P)** Representative immunofluorescence images (O) and quantitative analysis (P) of ACLY and ACSS2 levels in growing oocytes after *Acss2* knockdown (n=32, siNC; n=26, si*Acss2*). Nuclei were stained with DAPI (light blue). Scale bar =25 μm. **(Q-R)** Representative images (Q) and qualitative analysis (R) of the fluorescence intensity of growing follicles stained with anti-H3K27ac (violet) antibody from WT and *Acly* cKO mice (n=3). Nuclei were stained with DAPI (light blue). Scale bar = 25 μm. *NS* *p*>0.05; * *p*<0.05; *** *p*<0.001. Student’s t test (A, D, E, H, J, L, N, P, and R). F/F indicates *Acly*^flox/flox^ mice, and *Acly* cKO indicates *Acly^flox/flox^; Gdf9-Cre* mice.

**
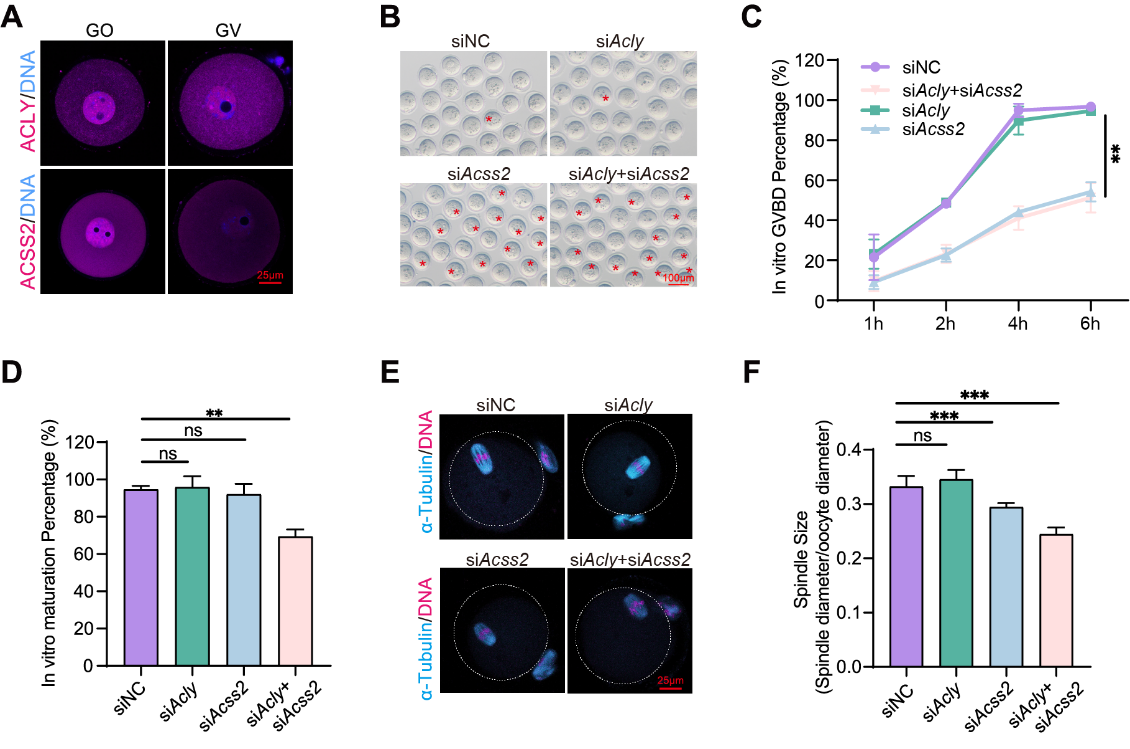
**

**Figure S11. Knocking down *Acss2* impairs oocyte maturation.**

1. Representative immunofluorescence (IF) images of ACLY and ACSS2 levels in growing oocytes (GO) and fully-grown oocytes at germinal vesicle stage (GV) (n = 15 oocytes per group). Nuclei were stained with DAPI (light blue). Scale bar = 25μm. Data are presented as mean ± S.D. **(B)** Bright-field images of germinal vesicle (GV) stage oocytes following siRNA-mediated knockdown (KD) of *Acly*, *Acss2*, or double KD (si*Acly*+si*Acss2*). Oocytes were maintained in M2 medium supplemented with milrinone for 8 h, followed by maturation in milrinone-free M2 medium for 16 h. Scale bar = 100 μm. **(C)** Germinal vesicle breakdown (GVBD) kinetics after milrinone release. Data are presented as mean ± S.D. n = 60 oocytes per group. **(D)** Polar body extrusion (PBE) rates after 16 h culture in M2 medium. **(E)** Representative immunofluorescence images of MII oocytes stained for α-tubulin (light blue) to visualize spindle morphology. Nuclei were stained with DAPI (violet). Scale bar = 25 μm. **(F)** Spindle size (diameter/oocyte diameter) measurements in MII oocytes (n = 15 spindles per group). Data are presented as mean ± S.D.** *p*<0.01; *** *p*<0.001. One-way ANOVA (C, D, and F).

**Supplementary Tables**

**Supplementary Table 1. Antibody information.**

| **Protein name** | **Manufacture** | **Catalogue number** |
| --- | --- | --- |

| ACLY | Abcam | Ab40793 |
| --- | --- | --- |
| ACSS2 | Abcam | Ab66038 |
| Fitc-α-Tubulin | Sigma | F2043 |
| α-Tubulin | CST | 2144S |
| β-actin | Proteintech | 66009-1-Ig |
| pPoI II S2 | Abcam | Ab5095 |
| NANOG | CST | 8822T |
| NANOG | Santa Cruz | 293121 |
| CDX2 | BioGenex | AM392 |
| CDX2 | Abcam | ab76541 |
| Histone H3 | Abcam | ab1791 |
| Acetyl-Histone H3 | Active Motif | 61937 |
| H3K4me3 | Abcam | Ab213224 |
| H3K27ac | Abcam | Ab177178 |
| H3K27ac | Active Motif | 39085 |
| H3K9ac | Active Motif | 91103 |
| H3K9me3 | Abcam | Ab8898 |
| H3K27me3 | CST | 9733S |
| H3K36me3 | Abcam | ab9050 |
| HDAC1 | Proteintech | 10197-1-AP |
| HDAC2 | CST | 57156 |
| HDAC3 | Proteintech | 10255-1-AP |
| P-HDAC2 (Ser394) | Affinity | AF3470 |
| Pan-Cr | PTM Bio | PTM-501 |
| Flag | Sigma | F1804 |
| HA | CST | 3724S |
| HAT1 | Santa Cruz | 390562 |
| KAT2A | Santa Cruz | 365321 |
| KAT2B | Santa Cruz | 13124 |
| P300 | Santa Cruz | 48343 |
| CBP | Santa Cruz | 7300 |
| DDB1 | CST | 6998S |
| pAKT (S473) | CST | 4060S |
| pACLY (S455) | CST | 4331 |
| YAP1 | Santa Cruz | sc-101199 |
| EZH2 | CST | 5246 |
| ADD3 | Affinity | DF3510 |
| ACTN1 | Affinity | BF8341 |
| FOXO3A | Proteintech | 66428-1-Ig |

**Supplementary Table 2. The sequence of PCR primers for genotyping.**

| Primer name | Target Gene | Application | Sequences (5′-3′) |
| --- | --- | --- | --- |
| *Acly* WT-F | *Acly* | Genotyping of WT/Flox allele | 5′- AGACCAGCGTAGAGGAGACACG -3′ |
| *Acly* WT-R |  |  | 5′- GCCAGTCCTTGTGGCCTAAGACT -3′ |
| *Acly*-KO-F |  | Genotyping of KO allele | 5′- AGACCAGCGTAGAGGAGACACG -3′ |
| *Acly*-KO-R |  |  | 5′- AAGTTAGCACACAGCCATCTGGC -3′ |
| *Gdf9*-F | *Gdf9* | Genotyping of Gdf9 Cre | 5′- GGTTTCTGTTGGGCTCTCAC -3′ |
| *Gdf9*-R |  |  | 5′- ATCAGAGGTGGCATCCACAG -3′ |

**Supplementary Table 3. The sequence of siRNAs.**

| siRNA name | Target Gene | Target Sequences (5′-3′) |
| --- | --- | --- |
| *siAcly* | *Acly* | GCTTCCTCAAGAACTTTCT |
| *siAcss2*-1 | *Acss2* | UGUACACCAGUGGCUCCAC |
| *siAcss2*-2 | *Acss2* | GGAUCAUGCGCACAGUCUA |
| *siAcss2*-3 | *Acss2* | AUUGCCACACCAGACUACA |
| si*P300*-1 | *P300* | CAGTTACCAGAACAGGTAT |
| si*P300*-2 | *P300* | CTCGATAGTGTTCATTTCT |
| si*P300*-3 | *P300* | CGTATTGTCCATGACTACA |
| *siHat1* | *Hat1* | UAUUCUCGAAAGCCUCUAC |

**Supplementary Table 4. Real-time PCR primer Sequence.**

| Primer name | Target Gene | Sequences (5′-3′) |
| --- | --- | --- |
| *Ercc*-F | *Ercc* | 5′- TCATTCGCCATAGAAGCCGAT -3′ |
| *Ercc*-R |  | 5′- TTCCCTGCGTTTGTATAGCCG -3′ |
| *Gapdh*-F | *Gapdh* | 5′- AGGTCGGTGTGAACGGATTTG -3′ |
| *Gapdh*-R |  | 5′- GGGGTCGTTGATGGCAACA -3′ |
| *Actin-*F | *Actin* | 5′- CCGTAAAGACCTCTATGCC -3′ |
| *Actin-*R |  | 5′- CTCAGTAACAGTCCGCCTA -3′ |
| *Acly-*F | *Acly* | 5′- CCAAGGCAATTTCAGAGCAGA -3′ |
| *Acly-*R |  | 5′- CAGAGAGAGATTGACCCCGAC -3′ |
| *Acss2-*F | *Acss2* | 5′- GTGAAAGGATCTTGGATTCCAGT -3′ |
| *Acss2-*R |  | 5′- CAGATGTTTGACCACAATGCAG -3′ |
| *Cdx2-*F | *Cdx2* | 5′- TACCCGGACTACGGTGGTTAC -3′ |
| *Cdx2-*R |  | 5′- GTGATGGTGCGCGTGGTAT -3′ |
| *Nanog-*F | *Nanog* | 5′- CACAGTTTGCCTAGTTCTGAGG -3′ |
| *Nanog-*R |  | 5′- GCAAGAATAGTTCTCGGGATGAA -3′ |
| *Hdac1-*F | *Hdac1* | 5′-AGTCTGTTACTACTACGACGGG-3′ |
| *Hdac1-*R |  | 5′-TGAGCAGCAAATTGTGAGTCAT-3′ |
| *Ezh2-*F | *Ezh2* | 5′-AGTGACTTGGATTTTCCAGCAC-3′ |
| *Ezh2-*R |  | 5′-AATTCTGTTGTAAGGGCGACC-3′ |
| *Yap1-*F | *Yap1* | 5′-TGAGATCCCTGATGATGTACCAC-3′ |
| *Yap1-*R |  | 5′-TGTTGTTGTCTGATCGTTGTGAT-3′ |
| *Foxo3-*F | *Foxo3* | 5′-GGGGAACCTGTCCTATGCC-3′ |
| *Foxo3-*R |  | 5′-TCATTCTGAACGCGCATGAAG-3′ |
| *Add3-*F | *Add3* | 5′-AGCAGAGGAAGCGAGTCACT-3′ |
| *Add3-*R |  | 5′-GCTTGGGTTGTGGCCTTTCT-3′ |
| *Actn1-*F | *Actn1* | 5′-GTCATCTCAGGTGAACGCTTG-3′ |
| *Actn1-*R |  | 5′-ACCACAGGAGTAACCCTTCTTT-3′ |
